# Novel role of NCoR1 in impairing spatial memory through the mediation of a novel interacting protein DEC2

**DOI:** 10.1007/s00018-024-05321-0

**Published:** 2024-06-20

**Authors:** Kuang-Min Cheng, Wei-Lun Hsu, Yun-Li Ma, Yen-Chen Liu, Eminy H. Y. Lee

**Affiliations:** 1 Graduate Institute of Life Sciences, National Defense Medical Center, Taipei, Taiwan; 2 Institute of Biomedical Sciences, Academia Sinica, Taipei, 115 Taiwan

**Keywords:** NCoR1, DEC2, C/EBPα, BDNF, Integrin α3, SGK1, Spatial memory, Y-maze learning

## Abstract

Long-term memory formation requires de novo RNA and protein synthesis. Using differential display PCR, we found that the *NCoR1* cDNA fragment is differentially expressed between fast learners and slow learners, with fast learners showing a lower expression level than slow learners in the water maze learning task. Fast learners also show lower *NCoR1* mRNA and protein expression levels. In addition, spatial training decreases both *NCoR1* mRNA and protein expression, whereas *NCoR1* conditional knockout (cKO) mice show enhanced spatial memory. In studying the molecular mechanism, we found that spatial training decreases the association between NCoR1 and DEC2. Both NCoR1 and DEC2 suppress the expression of BDNF, integrin α3 and SGK1 through C/EBPα binding to their DNA promoters, but overexpression of DEC2 in *NCoR1* cKO mice rescues the decreased expression of these proteins compared with *NCoR1 loxP* mice overexpressing DEC2. Further, spatial training decreases DEC2 expression. Spatial training also enhances C/EBPα binding to *Bdnf*, *Itga3* and *Sgk1* promoters, an effect also observed in fast learners, and both NCoR1 and DEC2 control C/EBPα activity. Whereas knockdown of BDNF, integrin α3 or SGK1 expression impairs spatial learning and memory, it does not affect Y-maze performance, suggesting that BDNF, integrin α3 and SGK1 are involved in long-term memory formation, but not short-term memory formation. Moreover, NCoR1 expression is regulated by the JNK/c-Jun signaling pathway. Collectively, our findings identify DEC2 as a novel interacting protein of NCoR1 and elucidate the novel roles and mechanisms of NCoR1 and DEC2 in negative regulation of spatial memory formation.

**Supplementary Information:**

The online version contains supplementary material available at 10.1007/s00018-024-05321-0.

## Introduction

It is well accepted that long-term memory formation requires de novo RNA and protein synthesis. Early studies showed that inhibition of mRNA and protein synthesis impairs long-term memory formation in different learning tasks in rats [1–3]. New protein synthesis and proper translational control were also found to be necessary for long-term memory formation [4]. Various strategies have been used to identify candidate genes associated with specific forms of learning and memory in different animals. For example, screening of *Drosophila* mutants has yielded about 10 genes that are associated with olfactory learning and memory [5]. Using a microarray analysis of the rat hippocampus, Cavallaro et al. identified 140 genes that are associated with water maze learning [6]. A similar analysis revealed 50 genes that are differentially expressed between superior learners and impaired learners in the same learning task in aged rats [7]. Using the differential display polymerase chain reaction (DD-PCR) method, we previously identified the *Iap* (integrin-associated protein) gene as being associated with memory formation of one-way inhibitory avoidance learning in rats [8]. We further showed that the *Thrp* (thyroid hormone responsive protein) gene is associated with the early phase of long-term potentiation (LTP) in the rat hippocampus [9]. Moreover, using the same method, we have identified 98 cDNA fragments from the rat dorsal hippocampus that are differentially expressed between fast learners and slow learners in the water maze learning task; one of these encodes the *Sgk1* (serum- and glucocorticoid-inducible kinase 1) gene [10]. We further demonstrated that SGK1 expression and activation of SGK1 signaling facilitates spatial memory and hippocampal LTP in rats [11, 12]. From the same analysis, we subsequently identified the *Pias1* (protein inhibitor of activated STAT1) gene and showed that PIAS1 expression enhances spatial memory in rats [13]. These results together support the idea that gene expression resulting from learning-associated neuronal activation plays an important role in long-term memory. More recently, we identified the *Ndfip1* (Nedd4 family interacting protein 1) gene in the same DD-PCR analysis, showing that NDFIP1 negatively regulates spatial memory formation through enhanced ubiquitination of certain target proteins [14]. This latter finding is consistent with the concept that removal of inhibitory synaptic constraints is equally important in long-term synaptic plasticity and long-term memory [15].

In addition to the *Sgk1*, *Pias1* and *Ndfip1* genes described above, our previous study identified other cDNA fragments that are also associated with spatial memory formation in rats [10]. One of these cDNA fragments encodes the *NCoR1* (nuclear receptor corepressor 1) gene – a major component of the NCoR complex [16, 17] and a corepressor for nuclear receptors [18]. NCoR1 and NCoR2 (also known as SMRT) are known to recruit and activate histone deacetylase 3 (HDAC3) and regulate gene expression through epigenetic modifications that mediate nuclear receptor signaling [19, 20]. But the mechanism of NCoR1 underlying spatial learning and memory is not known. In the present study, we aimed to examine the role of NCoR1 in spatial memory formation and the molecular mechanisms involved.

## Materials and methods

### Differential display PCR

We have performed differential display PCR (DD-PCR) in an earlier study by using 80 arbitrary random primers (H-AP1BH-AP80, RNAimage Kit purchased from GenHunter, Nashville, TN) to compare genes that are differentially expressed between the fast-learning rats (fast learners) and slow-learning rats (slow learners) from the water maze learning task [10]. The gene chosen for the present study is one of the cDNA fragments obtained earlier.

### Animals

Adult male Sprague–Dawley rats (230–300 g) were purchased from the BioLASCO, Taiwan. The *NCoR1*^*flox/flox*^ mice (strain name: *Ncor1*^*tm1Anh*^/J, stock number: 017632) and the wild-type mice (strain name: C57BL/6 J, stock number: 000664) were both purchased from Jackson Laboratory (Bar Harbor, ME). The *NCoR1*^*flox/flox*^ mice were mated and all animals were bred at the Animal Facility of the Institute of Biomedical Sciences, Academia Sinica, Taiwan. Mice of both sexes were used in the present study. They were maintained on a 12/12 h light/dark cycle (with light on at 8:00 am) with food and water continuously available. All experimental procedures adopted were in accordance with the "Guidelines of Animal Use and Care of the National Institute of Health" and were approved by the Animal Committee of Academia Sinica.

### Drugs and drug infusion to the hippocampus

SP600125 was purchased from Sigma-Aldrich (St. Louis, MO). It was first dissolved in 100% DMSO (Sigma-Aldrich) and further diluted with PBS to a final concentration of 1 µg/µl in 45% DMSO. N-methyl-D-aspartate (NMDA) was purchased from Tocris Bioscience (St. Louis, MO). It was dissolved in PBS at 8 mM concentration. Drugs were prepared immediately before use. The injection volume was 0.2 µl for each side of the CA1 area. The injection rate was 0.1 µl/min. Animals were sacrificed 3 h after SP600125 injection or 30 min after NMDA injection, and their hippocampal tissue was dissected out and stored at -80 °C until further biochemical assays.

### Plasmid DNA and small interference RNA (siRNA) transfection to the mouse hippocampus

The mouse Flag-*DEC2* plasmid (Product ID: OMu19590) was purchased from GenScript (Piscataway, NJ). To knockdown the expression of DEC2 in the mouse hippocampus, the *DEC2* siRNA was designed and used. The sequence for *DEC2* siRNA sense strand used was 5′-AAGAGAGACAGUUACUGGAACtt-3′ and the sequence for *DEC2* siRNA antisense strand used was 5′-GUUCCAGUAACUGUCUCUCUUtt-3′. To knockdown the expression of BDNF in the mouse hippocampus, the *BDNF* siRNA was designed and used. The sequence for *BDNF* siRNA sense strand used was 5′- GAGCGUGUGUGACAGUAUUtt-3′ and the sequence for *BDNF* siRNA antisense strand used was 5′-AAUACUGUCACACACGCUCtt-3′. To knockdown the expression of integrin α3 in the mouse hippocampus, the *integrin α3* siRNA was designed and used. The sequence for *integrin α3* siRNA sense strand used was 5′-AAGUGCGACAGCAACCUGCAGtt-3′ and the sequence for *integrin α3* siRNA antisense strand used was 5′-CUGCAGGUUGCUGUCGCACUUtt-3′. To knockdown the expression of SGK1 in the mouse hippocampus, the *SGK1* siRNA was designed and used. The sequence for *SGK1* siRNA sense strand used was 5′- AAGCAUUGCUGCUACAAAUAUtt-3′ and the sequence for *SGK1* siRNA antisense strand used was 5′-AUAUUUGUAGCAGCAAUGCUUtt-3′. The Silencer™ Negative Control number 1 siRNA (Thermo Fisher, Waltham, MA) was used as the control. Plasmid DNA and siRNA transfections were performed using the non-viral transfection agent polyethyleneimine (PEI), and we have previously shown that PEI does not produce toxicity to hippocampal neurons [21]. Before injection, Flag-*DEC2* plasmid DNA was diluted in 5% glucose to a stock concentration of 2.77 µg/µl. Branched PEI of 25 kDa (Sigma-Aldrich) was diluted to 0.1 mM concentration in 5% glucose and stored at 4 °C. Stock *DEC2* siRNA, *BDNF* siRNA, *integrin α3* siRNA and *SGK1* siRNA were diluted to a concentration of 30 nM in nuclease-free water (Sigma-Aldrich) and added to PEI at the ratio of 1:1. The DNA solution was mixed with 0.1 mM PEI consisting of 0.45 µl PEI and 0.55 µl plasmid DNA, resulting in a final concentration of 1.5 µg/µl for the plasmid DNA. The plasmid DNA or the siRNA mixture was subjected to vortex for 30 s and allowed to equilibrate for 15 min. For intra-hippocampal injection, 0.2 μl of Flag-*DEC2* plasmid or different siRNAs (15 pmol) were transfected to the mouse CA1 area bilaterally. The control group received Flag-vector or control siRNA transfection. The injection needle was left in place for 5 min to limit the diffusion of injected plasmid DNA and siRNA. Animals were sacrificed 48 h after plasmid DNA or siRNA injection and their hippocampal tissue was dissected out and stored at − 80 °C for biochemical assays. For the behavioral experiments, *DEC2* siRNA, *BDNF* siRNA, *integrin α3* siRNA, *SGK1* siRNA or control siRNA was also transfected to the mouse CA1 area bilaterally, and spatial learning starts 48 h after siRNA transfection. The retention test (probe trial) was performed the next day after the end of spatial learning. Animals were sacrificed after the probe trial test and their hippocampal tissue was dissected out for determination of DEC2 expression for the *DEC2* siRNA transfection experiment. For animals transfected with *BDNF* siRNA, *integrin α3* siRNA and *SGK1* siRNA, the Y-maze test was conducted 5 h after the probe trial test. These animals were sacrificed after the Y-maze test and their hippocampal tissue was dissected out for determination of BDNF, integrin α3 and SGK1 expression.

### Lenti-NLS-Cre construction and injection to the mouse hippocampus

The lenti-NLS-Cre vector construct was prepared according to that described previously [22]. For construction of the GFP-2A-NLS-Cre lentiviral vector, nuclear localization signal (NLS) was added to full-length Cre recombinase cDNA using PCR amplification and cloned into pLenti-Tri-cistronic (ABM, Richmond, BC, Canada) to obtain a bicistronic vector expressing both GFP and NLS-Cre. The primers used for Cre vector were: 5′-ATCGGAATTCCCAAAGAAGAAGAGAAAGGTTATGTCCAATTTACTGACC-3′ (forward) and 5′-ATCGGCGGCCGCCTAATCGCCATCTTCCAG-3′ (reverse). The PCR product was sub-cloned between the *EcoRI* and *NotI* sites of the lentiviral vector pLenti-Tri-cistronic (ABM). The GFP construct was cloned by amplifying the *GFP* gene from pLenti-CMV-GFP-2A-Puro-Blank (ABM) and sub-cloned into the pLenti-Tri-cistronic vector between *ScaI* and *KpnI* sites, upstream of the 2A peptide (a self-processing viral peptide bridge) and NLS-Cre sequences. The primers used for GFP vector were 5′-ATCGAGTACTGCCACCATGGAGATCGAGTGCCGCATC-3′ (forward) and 5′-ATCGGGTACCGGCGAAGGCGATGGGGGTC-3′ (reverse). For lentivirus packaging, HEK293LTV cells (Cell Biolabs, San Diego, CA) were transfected with 6 µg of psPAX2 (plasmid #12260, Addgene, Watertown, MA), 2 µg of pMD2.G (plasmid #12259, Addgene), and 8 µg of pLenti-GFP-2A-NLS-Cre (or 8 µg of pLenti-CMV-GFP-2A-Puro-Blank (ABM) coding for GFP as control) using 40 µl of Lipofectamine 2000 (Invitrogen, Carlsbad, CA) in 10 ml serum-free medium in a 10 cm cell culture dish. For lentiviral particle harvesting, the Lenti-X Concentrator (Takara Bio, Shiga, Japan) was used and prepared according to the manufacturer’s protocol. The transfection medium was removed from the cell culture dish 24 h after transfection and 12 ml of complete culture medium was added to the culture dish. Cell culture medium containing lentiviral particles was collected for three times at 24 h interval. The lentivirus-containing medium was pooled together and clarified by centrifugation at 500× g for 10 min. The clear supernatant was transferred to a fresh tube. The Lenti-X Concentrator (12 ml) was added to the clear supernatant (36 ml) and the mixture was incubated at 4° C for overnight. The mixture was then centrifuged at 1500× g for 45 min at 4 °C and the supernatant was removed. The pellet was re-suspended in 0.48 ml of ice-cold Dulbecco's Modified Eagle Medium (DMEM). The titer of the lentivirus was determined by using the Lentivirus qPCR Titer Kit (ABM). The re-suspended lentiviral vector was diluted by DMEM to a final concentration of 5 × 10^8^ IU/ml and stored at − 80 °C in aliquots.

For lentiviral vector injection, mice were anesthetized with pentobarbital (40 mg/kg, i.p.) and subjected to stereotaxic surgery without cannulation. Lentiviral vector was directly injected to their CA1 area bilaterally at the following coordinates: − 1.8 mm posterior to the bregma, ± 1.3 mm lateral to the midline, and − 2.1 mm ventral to the skull surface. A volume of 0.2 µl was injected to each side of the CA1 area. The infusion rate was 0.1 µl/min. Spatial learning started two weeks after lentiviral vector injection.

### Quantitative real-time PCR (Q-PCR)

Total RNA from rat CA1 tissue was isolated by using the RNAspin mini kit (GE Healthcare, Chicago, IL). Superscript III reverse transcriptase (Invitrogen) was used to generate the cDNA from total RNA. Real-time PCR analysis was conducted by using the ABI PRISM 7500 real-time PCR system with *Power* SYBR Green PCR Master Mix (Applied Biosystems, Foster City, CA). The primer sequences used for *NCoR1* were: 5′-TGTGTCAGCAGCACCTTTAGA-3′ (forward) and 5′-GCTCGACTAGGAGGGCTTTC-3′ (reverse). The primer sequences used for *HRPT* were: 5′-GCCGACCGGTTCTGTCAT-3′ (forward) and 5′-TCATAACCTGGTTCATCATCACTAATC-3′ (reverse). The cycle threshold (*Ct*) value and data were analyzed by using the 7500 system Sequence Detection Software (Applied Biosystem). Quantification of *NCoR1* mRNA expression was normalized to that of *HPRT* mRNA expression.

### Cell culture

The primary mouse cortical neurons were purchased from Gibco (Thermo Fisher, MA). The primary neurons were maintained in Neurobasal/B-27 medium (Thermo Fisher) containing 0.5 mM glutamine. The mouse Neuro2A neuroblastoma cells were maintained in HyClone Dulbecco’s modified Eagle’s medium (DMEM) (Cytiva, MA) supplemented with 10% fetal bovine serum (Thermo Fisher). All the cells were maintained in a humidified 5% CO_2_-95% air atmosphere at 37 °C.

### Luciferase reporter assay

For construction of the pGL4.10 luciferase reporter plasmid containing different gene promoters, the 1.2 kb length *Bdnf* promoter, the 1.2 kb length *Itga3* promoter and the 1.2 kb length *Sgk1* promoter were cloned and amplified from the mice hippocampal genomic DNA with primers 5′-ATGCGGTACCGCATGGCCTTGGGAACAAGT-3′ and 5′- ATGCGATATCAGTGGCTGCTTCAAGGTTCA-3′ (for *Bdnf* promoter), primers 5′-ATGCGGTACCACCGGAGCAGAATCCCATTG-3′ and 5′-ATGCGATATCTCCGTTTTCCAGATCCGTGG-3′ (for *Itga3* promoter) and primers 5′-ATGCGGTACCGTGGCGTCGGATGTTACAGA-3′ and 5′-ATGCGATATCGCAGCAAACCACAGGGTAGA-3′ (for *Sgk1* promoter), respectively. The PCR products were sub-cloned between the KpnI and EcorV sites of the pGL4.10 [*luc2*] vector (Promega, Madison, WI). Neuro2A cells were maintained in Dulbecco's modified Eagle’s medium containing 10% fetal bovine serum and incubated at 37 °C in a humidified atmosphere with 5% CO_2_. For luciferase reporter assay, different gene promoter of the pGL4.10 luciferase reporter plasmid (0.8 μg) and pGL4.74 plasmid (0.2 μg) were co-transfected with *DEC2* siRNA (or control siRNA) (20 pmol) to Neuro2A cells and primary mouse cortical neurons at DIV 5 using Lipofectamine™ 2000 (4 μl) (Thermo Fisher). Luciferase activity assay was performed 48 h later using the Dual luciferase reporter assay system (Promega) and the TD-20/20 Luminometer (Turner Designs Hydrocarbon Instruments, Fresno, CA). The relative activity was normalized by Renilla luciferase activity.

### Immunoprecipitation and western blot

Immunoprecipitation (IP) and Western blot for hippocampal tissue lysate were performed according to that described previously [23]. Briefly, rat CA1 tissue was lysed by brief sonication in RIPA lysis buffer containing 50 mM Tris–HCl (pH 7.4), 150 mM NaCl, 2 mM EDTA, 1% IGEPAL CA-630 and 20 mM N-ethylmaleimide (Catalog No. E3876-5G, Sigma-Aldrich). One tablet of protease inhibitor cocktail (cOmplete ULTRA Tablets, Mini, EDTA-free, EASYpack, Roche, Mannheim, Germany) and one tablet of phosphatase inhibitor (PhosSTOP, Roche) were added to each 10 ml of the RIPA lysis buffer. For IP of NCoR1, the clarified lysate (0.5 mg) was immunoprecipitated with 3 µl of anti-NCoR1 antibody (ProteinTech, Rosemont, IL), 15 µl of Protein G Mag Sepharose Xtra beads (Sigma-Aldrich) and 15 µl of Protein A Mag Sepharose Xtra beads (Sigma-Aldrich) at 4 °C overnight. The immune complex on beads were washed three times with 1X PBS and heated in NuPAGE™ LDS Sample Buffer (Thermo Fisher) at 70 °C for 10 min and were subjected to 7% Tris–Acetate gel followed by transferring onto the PVDF membrane (Millipore, Bedford, MA). Western blot was conducted using the following antibodies: anti-DEC2 (1:10,000; Abcam, Cambridge, UK), anti-NCoR1 (1:2000; Bethyl Laboratories, Montgomery, TX), anti-BDNF (1:1000, Thermo Fisher), anti-JNK (1:1000, Santa Cruz), anti-p-JNK (1:2000, Cell Signaling), anti-c-Jun (1:2000, Cell Signaling), anti-p–c-Jun (1:1000, Cell Signaling), anti-integrin α3 (1:2000, Sigma-Aldrich), anti-SGK1 (1:2000, Sigma-Aldrich) and anti-actin (1:200,000; Millipore) antibodies. The secondary antibody used was HRP-conjugated goat-anti rabbit IgG light chain antibody (1:8000, Jackson ImmunoResearch, West Grove, PA) or HRP-conjugated goat-anti mouse IgG light chain antibody (1:8000, Jackson ImmunoResearch). Membrane was developed by reacting with chemiluminescence HRP substrate (Millipore) and was exposed to the LAS-3000 image system (Fujifilm, Tokyo, Japan) for visualization of protein bands. The protein bands were quantified by using the NIH Image J Software.

### Chromatin immunoprecipitation (ChIP) assay

ChIP assay was performed according to the protocol described in the Millipore ChIP assay kit. The hippocampal tissue from *DEC2* siRNA and control siRNA-transfected mice and the hippocampal tissue from *NCoR1* cKO and *NCoR1 loxP* mice were washed using ice-cold PBS and fixed with 1% formaldehyde by adding formaldehyde to the ice-cold PBS for 10 min. After adding glycine to quench the un-reacted formaldehyde, tissues were homogenized and re-suspended in cell lysis buffer plus protease inhibitor cocktail II, then switched to nuclear lysis buffer plus protease inhibitor cocktail II for sonication. The chromatin was immunoprecipitated with 3 µl rabbit anti-C/EBPα antibody (GeneTex, Irvine, CA), 15 µl each of Protein G Mag Sepharose Xtra beads and Protein A Mag Sepharose Xtra beads (Sigma-Aldrich) at 4 °C for overnight. DNA purified from the immunoprecipitated samples was subjected to PCR reaction. For examination of mouse C/EBPα binding to the *Bdnf* gene promoter, the forward primer used for the mouse *Bdnf* promoter was: 5′-AGGTCAAATGCCATCTCCTCA-3′ (nucleotide − 2622 to − 2602) and the reverse primer was: 5′-AGACAGCACAGGATTGTACTGAA-3′ (nucleotide − 2442 to − 2464). The PCR product for the mouse *Bdnf* promoter is 181 bps in length. For examination of mouse C/EBPα binding to the *Itga3* gene promoter, the forward primer used for the mouse *Itga3* promoter was: 5′-CATTCCAGGCAGCCGAACA-3′ (nucleotide − 788 to − 770) and the reverse primer was: 5′-GCTTCTTCCTAGGAGCGTCG-3′ (nucleotide − 596 to − 615). The PCR product for the mouse *Itga3* promoter is 193 bps in length. For examination of mouse C/EBPα binding to the *Sgk1* gene promoter, the forward primer used for the mouse *Sgk1* promoter was: 5′-TCCTTGGGATGTTTTCGGTT-3′ (nucleotide − 1578 to − 1559) and the reverse primer was: 5′-TAGCGTTCATAAGCTCCGGC-3′ (nucleotide − 1400 to − 1419). The PCR product for the mouse *Sgk1* promoter is 179 bps in length. For examination of rat C/EBPα binding to the *Bdnf* gene promoter, the primer used for the rat *Bdnf* promoter was the same as the mouse primer. The forward primer was: 5′-AGGTCAAATGCCATCTCCTCA-3′ (nucleotide − 3076 to − 3056) and the reverse primer was: 5′-AGACAGCACAGGATTGTACTGAA-3′ (nucleotide − 2898 to − 2915). The PCR product for the rat *Bdnf* promoter is 179 bps in length. For examination of rat C/EBPα binding to the *Itga3* gene promoter, the forward primer used for the rat *Itga3* promoter was: 5′-TCCACCCCTTCACCTGGTAT-3′ (nucleotide − 1119 to − 1100) and the reverse primer was: 5′-TATTTCTCCTGTCCACCCGC-3′ (nucleotide − 926 to − 945). The PCR product for the rat *Itga3* promoter is 194 bps in length. For examination of rat C/EBPα binding to the *Sgk1* gene promoter, the forward primer used for the rat *Sgk1* promoter was: 5′-CATTTCGTGGGGAAGAAAGGG-3′ (nucleotide − 2134 to − 2114) and the reverse primer was: 5′-AACACCAGAGAAACTCAAAGGG-3′ (nucleotide − 1946 to − 1925). The PCR product for the rat *Sgk1* promoter is 210 bps in length. In another experiment with SP600125 and DMSO injection to the mouse hippocampus, the procedures used for ChIP assay were the same as that described above except that the chromatin was immunoprecipitated with 3 µl rabbit anti-c-Jun antibody (Cell Signaling) for examination of c-Jun binding to the *NCoR1* gene promoter. The forward primer used for the *NCoR1* promoter was: 5′-TCGTTCCGCTGAGTTCCAAA-3′ (nucleotide − 1236 to − 1217) and the reverse primer was: 5′-GTTTCCACGCTAGACCACGA-3′ (nucleotide − 1006 to − 1025). The PCR product for the *NCoR1* promoter is 231 bps in length. The PCR products were separated by 2% agarose gel electrophoresis.

### Immunohistochemistry

The *NCoR1 loxP* and *NCoR1* cKO mice were anesthetized with pentobarbital (100 mg/kg, i.p.) and perfused with pre-cold PBS followed by 4% paraformaldehyde. Brains were removed and post-fixed in 20% sucrose/4% paraformaldehyde solution for 20–48 h. Frozen brains were cut into 30-µm sections on a cryostat and mounted on gelatin-coated slides. Brain sections were rinsed with 1 X TBS for 10 min followed by antigen retrieved with 0.1 M citric acid/0.1 M sodium citrate buffer at 95 °C for 45 min. The sections were washed with 1 X TBS for 10 min and permeabilization with 1 X TBST (0.5% Triton X-100 in 1 X TBS) for 10 min. Sections were pre-incubated in blocking solution (3% BSA and 0.5% Triton X-100 in 1 X TBS) for 1 h. EGFP fluorescence (green) was observed in the hippocampus of *NCoR1 loxP* mice transfected with lenti-GFP vector or lenti-GFP-Cre vector. For immunohistochemistry of NCoR1, brain sections were incubated with rabbit-anti-NCoR1 antibody (1:100, ProteinTech) at 4 °C for overnight. Brain sections were then washed with 1 X TBS and incubated with goat anti-rabbit secondary antibody conjugated with Alexa Fluor^®^ 594 (1:500, Jackson ImmunoResearch) for 1 h. For visualization of the nucleus, sections were mounted on slides with 15 µl of the DAPI Fluoromount-G^®^ mounting medium (SouthernBiotech, Birmingham, AL) and washed by 1 X TBS for 10 min for three times and stored at 4 °C. Photomicrographs were taken using a Zeiss LSM700 Stage confocal microscope.

### Immunofluorescence

Primary mouse hippocampal neurons and cultured Neuro2A cells were transfected with EGFP-vector plasmid DNA for 48 h on poly-L-lysine-coated glass coverslips. Cells were washed with PBS for 5 min and fixed with 4% paraformaldehyde for 10 min at room temperature followed by PBS wash for 10 min for three times. For immunofluorescence detection of the nucleus, cells on glass coverslips were added with 10 μl of the DAPI Fluoromount-G mounting medium. Photomicrographs were taken using a Zeiss LSM700 confocal microscope (Carl Zeiss, BW, Germany).

### Water maze learning

The water maze used in the present study was a plastic, circular pool, 1.2 m in diameter and 25 cm in height that was filled with water (25 ± 2 °C) to a depth of 16 cm. A circular platform of 10 cm in diameter was placed at a specific location away from the edge of the pool. The top of the platform was submerged 0.6 cm below the water surface. Water was made cloudy by adding milk powder. Distinctive, visual cues were set on the wall. For spatial acquisition, animals were subjected to three trials a day (as one session) with one given early in the morning, one given in the early afternoon and the other one given in the late afternoon. The acquisition procedure lasted for 5 days (for 5 sessions) and a total of 15 trials were given. For these trials, animals were placed at different starting positions spaced equally around the perimeter of the pool in a random order. Animals were given 60 s to find the platform. If an animal could not find the platform within 60 s, it was guided to the platform and was allowed to stay on the platform for 20 s. The time that each animal took to reach the platform was recorded as the escape latency. A probe trial of 60 s was given on day 6 to test their memory retention. Animals were placed in the pool with the platform removed and the time they spent in each quadrant (target quadrant, left quadrant, opposite quadrant and right quadrant), the total distance travelled in the target quadrant and their swim speed were recorded. For spatial training, the procedures used were the same as that for spatial acquisition except that training lasted for two consecutive days (for *NCoR1* mRNA measure) or three consecutive days (for NCoR1 protein measure). Animals were sacrificed at the end of the last training trial. For the water maze experiment with *DEC2* siRNA transfection, spatial learning started two days after siRNA transfection. To avoid degradation of the transfected siRNA, we have shortened the training period. Animals were subjected to four trials a day and spatial training lasted for two days (8 trials) only. Animals were similarly subjected to the probe trial test one day later and the same behavioral measures were recorded.

For screening of the fast-learning rats and slow-learning rats, the same criteria used in a previous study [10] were adopted here. Briefly, animals that reached the escape latency less than 30 s by the end of the third training session were designated as the fast-learning rats (fast learners). Animals that did not reach this escape latency until the end of the seventh training session were designated as the slow-learning rats (slow learners).

Animals were subjected to visible platform learning three days after the retention test. For the visible platform learning experiment, a flag was mounted on the platform and the platform was 2.5 cm above the water surface so the animals can visualize the flag and identify the location of the platform. In addition, milk powder was not added to the swimming pool, so the pool was not cloudy.

### Y-maze test

The Y-maze test was used to assess short-term spatial memory in mice as previously described [24, 25]. The Y-maze apparatus consists of three opaque identical arms (30 cm × 8 cm × 25 cm, length × width × height) labeled as arm A, arm B and arm C, respectively, with 120° angles apart from each other. In this study, spontaneous alternations were measured. Each animal was placed at the distal part of arm A facing towards the center of the maze, and was allowed to explore all three arms for 8 min. An alternation is defined as consecutive entries into all three arms. The number of total arm entries and alternations were recorded and the percentage of alternation is calculated as [the number of alternations/the total number of arm entries-2] × 100.

### Locomotor activity measure

Locomotor activity was measured in the Digiscan Animal Activity Monitor System as that described previously [26]. The activity chamber is 16 inches in square with 16 × 16 horizontal by vertical infrared sensors, and it is divided to nine sub-regions with equal size. These sensors were used to localize the animal's floor position. The number of crossovers between any two sub-regions and the speed of movement in the activity chamber for each *NCoR1 loxP* and *NCoR1* cKO mouse was recorded during the 20 min measurement period.

### Statistical analysis

Spatial acquisition data were analyzed with two-way analysis of variance (ANOVA) with repeated measure followed by post-hoc Newman-Keuls multiple comparisons (represented by q value). Retention performance data (time spent in the target quadrant and target quadrant distance travelled), the Y-maze performance, locomotor activity data and all biochemical data were analyzed with the Student’s t-test. Values of *p* < 0.05 were considered statistically significant (^*^ *p* < 0.05, ^**^ *p* < 0.01, ^***^ *p* ≤ 0.001).

## Results

### Identification of the *NCoR1* gene through DD-PCR screening

**Fig. 1:**
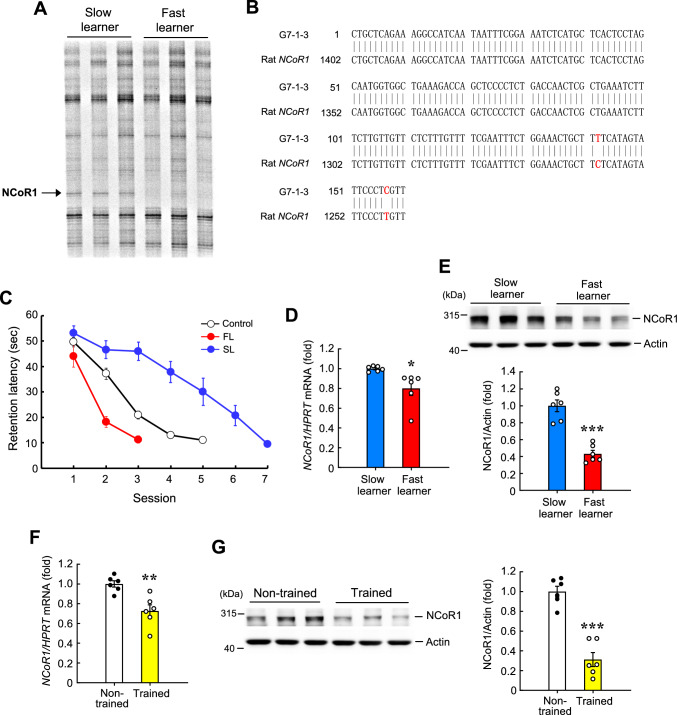
Identification of the *NCoR1* gene from water maze learning and NCoR1 expression is decreased by spatial training. **A** DD-PCR of hippocampal RNA associated with water maze learning in rats. One cDNA fragment (*NCoR1*) that is differentially expressed between the fast learners and slow learners is indicated by the arrow. **B** Alignment of the sequence of G7-1–3 (the arbitrary primers used) with the rat *NCoR1* gene. Letters marked in red indicate non-consensus nucleotides. **C** Water maze performance of fast learners (N = 6), slow learners (N = 6) and control rats (N = 23). FL: fast learner, SL: slow learner. **D** *NCoR1* mRNA level in the CA1 area of slow learners and fast learners (t_1,10_ = 2.8, *p* < 0.05). **E** NCoR1 protein level in the CA1 area of slow learners and fast learners (t_1,10_ = 8.4, *p* < 0.001). **F** *NCoR1* mRNA level in the CA1 area from non-trained (swimming control) and trained rats (2-day training) (t_1,10_ = 3.87, *p* < 0.01). **G** NCoR1 protein level in the CA1 area from non-trained and trained rats (3-day training) (t_1,10_ = 7.75, *p* < 0.001). N = 6 each group. Data are expressed as individual values and mean ± SEM or expressed as mean ± SEM (for C). * *p* < 0.05, ** *p* < 0.01 and *** *p* < 0.001

Using DD-PCR, we previously identified 98 cDNA fragments that are differentially expressed in the dorsal hippocampus between fast learners and slow learners in the water maze learning task [10]. Using the primer set H-A33 (5'‐end primer sequence, 5′-AAGCTTGCTGCTC-3′) and H-T11A (3'‐end primer sequence, 5′-AAGCTTTTTTTTTTTA-3′), we identified one 160-bp cDNA fragment (designated G7-1–3) that showed 98.8% sequence homology to the 3′-end region of the rat *NCoR1* gene (data accession number for *NCoR1*: NM_001271103.1) (Fig. 1A and B). This cDNA fragment was among the genes categorized as ‘unknown’ in our previous study [10]. The expression level of this gene was higher in the dorsal hippocampus of slow learners than fast learners (Fig. 1A).

### Spatial training decreases NCoR1 expression in the hippocampus

After identifying the *NCoR1* cDNA fragment as being differentially expressed between fast and slow learners, we examined the role of NCoR1 in spatial learning. To this end, we screened another batch of rats using the same criteria and procedures adopted previously [10] and obtained a new set of slow learners and fast learners (Fig. 1C). We examined *NCoR1* mRNA levels in one side of the CA1 area of these animals using Q-PCR. These analyses revealed that *NCoR1* mRNA levels were lower in fast learners than slow learners (Fig. 1D). The other side of the CA1 tissue from the same animals was subjected to Western blot analysis. We found that NCoR1 protein levels were significantly lower in fast learners than slow learners (Fig. 1E). These results suggest that NCoR1 expression is negatively associated with spatial acquisition. Based on these observations, we hypothesized that spatial training decreases NCoR1 expression. To test this hypothesis, we divided a different batch of rats into trained and non-trained groups. In the above experiments on *NCoR1* mRNA and protein expression in fast learners and slow learners, the animals swam in different sessions and spent different times in the learning task. To exclude the possibility that these differences affect NCoR1 expression level, subsequent spatial training experiments were designed such that animals spent the same amount of time during the task. Animals in the trained group received regular water maze training for 2 d for *NCoR1* mRNA measurements (time interval when the difference was most apparent) or 3 d for NCoR1 protein measurements (Western blot). Each animal in the non-trained group swam for the same amount of time as trained animals, except visual cues and platform were removed. The mean value from all trained animals was taken for a given trial. For example, if the mean value was 45 s for the third trial of the trained animals, each animal in the non-trained group was allowed to swim for 45 s in the third trial. Animals in the non-trained group were removed from the pool when the mean value time for each given trial was reached. Thus, the non-trained animals served as a swimming control group. Animals were sacrificed at the end of training (or swimming) and their CA1 tissue was dissected out for *NCoR1* mRNA and protein determinations. Results of these determinations revealed that spatial training decreased both *NCoR1* mRNA level (Fig. 1F) and NCoR1 protein expression (Fig. 1G) to an extent comparable to that in fast learners.

### Spatial learning and memory performance is enhanced in *NCoR1* conditional knockout (cKO) mice

**Fig. 2:**
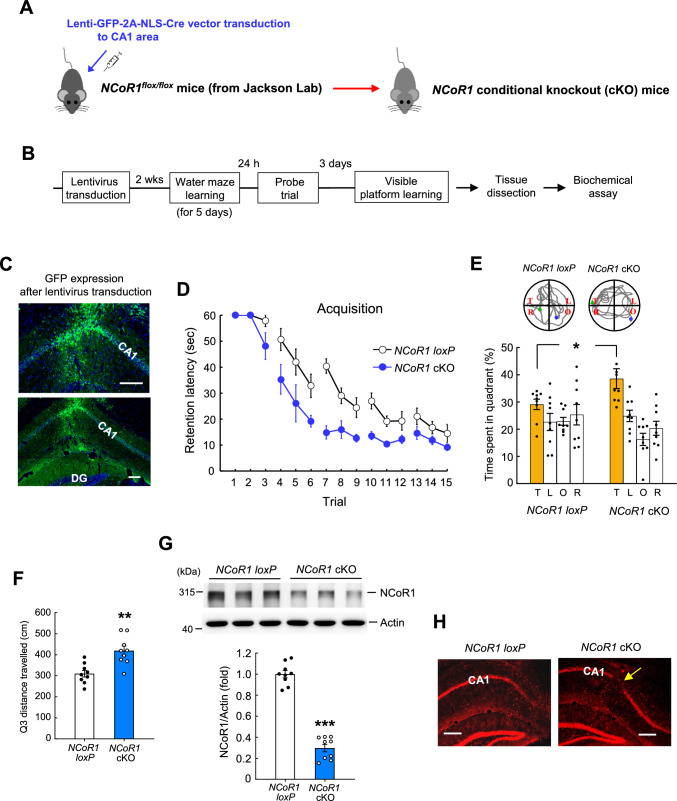
Spatial learning and memory is enhanced in *NCoR1* cKO mice. **A** Lenti-GFP-2A-NLS-Cre vector was transduced to the CA1 area of *NCoR1*^*flox/flox*^ mice to generate the *NCoR1* conditional knockout (cKO) mice. Lenti-GFP-vector was transduced to the CA1 area of *NCoR1*^*flox/flox*^ mice to generate the *NCoR1 loxP* control mice. **B** Schedule of lentiviral vector transduction to *NCoR1*^*flox/flox*^ mice, behavioral measure and biochemical assay. **C** Immunohistochemical staining of EGFP (green color) showing the location and expression of the injected lentivirus in the mouse CA1 area. DAPI staining is shown in blue color. Scale bar equals 100 µm for both the upper panel and lower panel. DG: dentate gyrus. **D** Acquisition performance of water maze learning from *NCoR1 loxP* mice and *NCoR1* cKO mice (F_1,16_ = 26.3, *p* < 0.001). **E** Probe trial performance (time spent in the target quadrant, t_1,16_ = 2.28, *p* < 0.05) and representative swim patterns from *NCoR1 loxP* mice and *NCoR1* cKO mice. **F** Distance travelled in the target quadrant for the probe trial test of *NCoR1 loxP* mice and *NCoR1* cKO mice (t_1,16_ = 3.99, *p* = 0.001). **G** NCoR1 expression level in *NCoR1 loxP* mice and *NCoR1* cKO mice after visible platform learning (t_1,16_ = 14.21, *p* < 0.001). N = 9 each group. **H** Immunohistochemical staining of NCoR1 (red color) showing decreased expression of NCoR1 in the CA1 area only of *NCoR1* cKO mice (indicated by the arrow). Scale bar equals 200 µm. Data are expressed as individual values and mean ± SEM or expressed as mean ± SEM (for **D**). * *p* < 0.05, ** *p* < 0.01 and *** *p* < 0.001

To further examine the role of NCoR1 in spatial learning and memory formation, we randomly divided *NCoR1*^*flox/flox*^ mice into two groups, transducing one with lenti-GFP-vector in the CA1 area to generate *NCoR1 loxP* control mice, and the other with lenti-GFP-2A-NLS-Cre-vector to generate *NCoR1* cKO mice (Fig. 2A). These mice were subjected to water maze learning, retention test, and visible platform learning according to the schedule shown in Fig. 2B. Immunohistochemical staining results for GFP showing the location and expression of the injected lentivirus are illustrated in Fig. 2C. These results revealed that *NCoR1* cKO mice showed enhanced acquisition performance compared with *NCoR1 loxP* mice (Fig. 2D). In addition, *NCoR1* cKO mice spent more time in the target quadrant (Fig. 2E) and traveled more extensively in the target quadrant (Fig. 2F) during the probe trial test than *NCoR1 loxP* mice. Further analyses indicated that the swim speed of these two groups of mice was similar (Fig. S1A) and their performance in visible platform learning was also not different (Fig. S1B). These animals were sacrificed after visible platform learning and their CA1 tissue was dissected out for Western blot determination of NCoR1 expression. Results showed that NCoR1 expression levels were significantly decreased in *NCoR1* cKO mice compared with *NCoR1 loxP* mice (Fig. 2G). Further immunohistochemical results confirmed that the decreased expression of NCoR1 in *NCoR1* cKO mice was specifically localized to the CA1 area (Fig. 2H).

In a different batch of animals, we further examined whether there is an alteration in motor function in *NCoR1* cKO mice that might affect their water maze performance. Locomotor activity of *NCoR1 loxP* mice and *NCoR1* cKO mice was measured in an activity chamber for 20 min 2 wk after lentiviral transduction. Results revealed that *NCoR1 loxP* and *NCoR1* cKO mice show similar performance in terms of the number of crossovers and speed of movement in the activity chamber (Fig. S1C). Further Western blot results indicated that NCoR1 expression levels were markedly decreased in *NCoR1* cKO mice compared with *NCoR1 loxP* mice (Fig. S1D).

### Spatial training decreases the association between NCoR1 and DEC2, and BDNF expression is increased in *NCoR1* cKO mice and *DEC2* siRNA-transfected mice

**Fig. 3:**
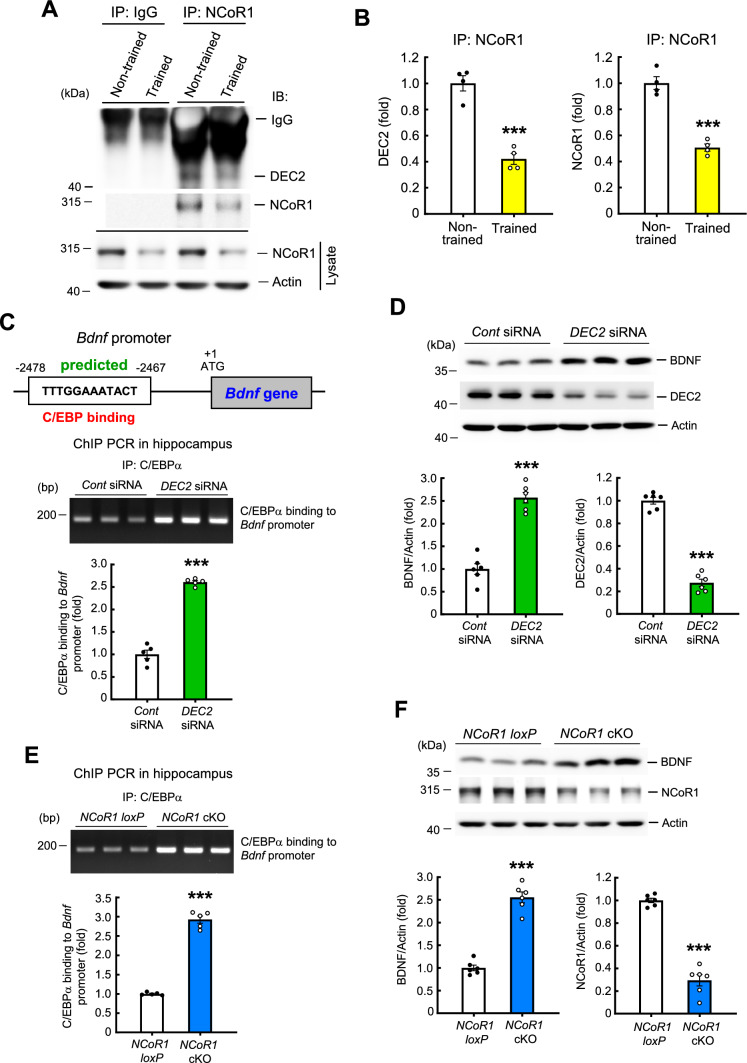
Spatial training decreases the association between NCoR1 and DEC2, and BDNF expression is increased in *DEC2* siRNA-transfected mice and in *NCoR1* cKO mice. **A** The CA1 tissue lysates from non-trained and trained rats were immunoprecipitated with anti-NCoR1 antibody and immunoblotted with anti-DEC2 antibody and anti-NCoR1 antibody. The same tissue lysates were also immunoprecipitated with IgG and immunoblotted with anti-DEC2 antibody and anti-NCoR1 antibody serving as the control experiment. The same lysates were also subjected to Western blot determination of NCoR1 expression (lower panel). N = 4 each group. **B** The quantified result of (**A**) from four independent experiments (t_1,6_ = 15.25, *p* < 0.001). **C** Promoter analysis prediction shows that the mouse *bdnf* DNA promoter contains the C/EBP binding element (upper panel). ChIP PCR assay showing C/EBPα binding to the *bdnf* promoter in the hippocampus from control siRNA- and *DEC2* siRNA (15 pmol)-transfected mice. The results were obtained from five independent experiments (t_1,8_ = 16.11, *p* < 0.001) (lower panel). **D** BDNF and DEC2 expression levels in the hippocampus from mice that received control siRNA or *DEC2* siRNA (15 pmol) transfection (t_1,10_ = 9.43, *p* < 0.001 for BDNF and t_1,10_ = 16.52, *p* < 0.001 for DEC2). N = 6 each group. **E** ChIP PCR assay showing C/EBPα binding to the *bdnf* promoter in the hippocampus of *NCoR1 loxP* mice and *NCoR1* cKO mice. The results were obtained from five independent experiments (t_1,8_ = 20.07, *p* < 0.001). **F** BDNF and NCoR1 expression levels in the hippocampus of *NCoR1 loxP* mice and *NCoR1* cKO mice (t_1,10_ = 8.93, *p* < 0.001 for BDNF and t_1,10_ = 13.47, *p* < 0.001 for NCoR1). N = 6 each group. IP: immunoprecipitation. Data are expressed as individual values and mean ± SEM. *** *p* < 0.001

The above results demonstrate that NCoR1 negatively regulates spatial learning and memory formation. Next, we examined the molecular mechanism underlying this regulation. As described above, NCoR1 is a major component of the NCoR complex [16, 17] and a corepressor for nuclear receptors [18]. But NCoR1 may also serve as a corepressor of other transcription factors in addition to nuclear receptors. DEC2 is a transcriptional repressor of members of the basic helix-loop-helix (bHLH) family [27] and is regulated by a molecular clock system [28]. DEC2 is also a clock protein that regulates sleep [29]. Further, memory performance is enhanced in mice lacking both DEC2 and DEC1 [30]. Because NCoR1 and DEC2 function as a corepressor and repressor, respectively, and because NCoR1 and DEC2/DEC1 both negatively regulate memory, we examined whether NCoR1 impairs spatial learning and memory through its interaction with DEC2. A co-immunoprecipitation (co-IP) experiment was first conducted to examine this issue. CA1 tissue lysates from non-trained and trained rats were immunoprecipitated with anti-NCoR1 antibody and immunoblotted with anti-DEC2 and anti-NCoR1 antibodies. For the control experiment, the same tissue lysates were immunoprecipitated with IgG and immunoblotted with anti-DEC2 and anti-NCoR1 antibodies. Results indicated that NCoR1 was associated with DEC2 in non-trained animals, but this association was markedly decreased in animals subjected to spatial training (Fig. 3A and B), probably owing to reduced availability of NCoR1 resulting from training. No specific band was observed following the immunoprecipitation of tissue lysates with IgG (Fig. 3A). On the other hand, NCoR1 expression levels in lysates were consistently decreased in trained animals compared with non-trained controls (Fig. 3A, lower panel).

Learning is known to activate NMDA receptor-mediated signaling [31]. To further confirm the effect of spatial training on alterations in the association between NCoR1 and DEC2, we acutely injected PBS or NMDA (8 mM) into the CA1 area in a different batch of rats. Rats were sacrificed 1 h later, and their CA1 tissue was subjected to the same co-IP experiment described above. Results revealed that NMDA administration similarly decreased the association between NCoR1 and DEC2 (Fig. S2A, right panel, and Fig. S2B). No specific band was observed when the same lysates were immunoprecipitated with IgG and immunoblotted with anti-DEC2 antibody (Fig. S2A, left panel). As expected, NCoR1 expression levels in lysates were decreased in NMDA-injected animals compared with PBS-injected animals (Fig. S2A, lower panels). Because NCoR1 is a component of the NCoR complex containing HDAC3, we also examined the relationship between NCoR1 and HDAC3 in the context of spatial training. CA1 tissue lysates from trained and non-trained rats were immunoprecipitated with anti-HDAC3 antibody and immunoblotted with anti-NCoR1 antibody. Results revealed that the association of NCoR1 with HDAC3 was similarly decreased in trained animals than non-trained animals. No specific band was observed when tissue lysates were immunoprecipitated with IgG and immunoblotted with anti-NCoR1 antibody (Fig. S2C and D, left panel). We further examined whether spatial training alters HDAC3 expression in the CA1 area. Results indicated that HDAC3 expression levels in lysates were similar in trained and non-trained animals (Fig. S2C, left panel).

DEC2 was reported to regulate C/EBPα binding to DNA promoters to inhibit adipogenic differentiation [32]. Brain-derived neurotrophic factor (BDNF) plays a critical role in mammalian learning and memory formation [33–35], and the *Bdnf* gene promoter is predicted to contain a C/EBP binding element (https://www.ncbi.nlm.nih.gov/genome/gdv/ from the NCBI genome browser, and http://alggen.lsi.upc.es/cgi-bin/promo_v3/promo/promoinit.cgi?dirDB=TF_8.3 from ALGGEN—PROMO) (Fig. 3C, upper panel). Here, we examined whether DEC2 downregulates BDNF expression through suppression of C/EBPα-mediated *Bdnf* gene expression. Mice were randomly divided into two groups and received control siRNA or *DEC2* siRNA (15 pmol) in their CA1 area via transfection. Mice were sacrificed 2 d later and their hippocampal tissue containing the CA1 area was dissected out and subjected to ChIP PCR assays for determination of C/EBPα binding to the *Bdnf* promoter. Results revealed that *DEC2* siRNA transfection significantly increased C/EBPα binding to the *Bdnf* promoter compared with control siRNA transfection (Fig. 3C). Because *DEC2* siRNA increased C/EBPα binding to the *Bdnf* promoter, it is expected that *DEC2* siRNA would increase BDNF protein expression. We examined this issue using Western blotting, which revealed that *DEC2* siRNA transfection markedly increased BDNF expression level in the hippocampus. DEC2 expression level was decreased (Fig. 3D), confirming the effectiveness of *DEC2* siRNA transfection. Because *DEC2* siRNA increased BDNF expression, and BDNF is critical for learning and memory formation, we expected that spatial training would decrease DEC2 expression. Consistent with this prediction, we found that DEC2 expression level was significantly lower in trained animals compared with non-trained controls (Fig. S2E and F). To further study the role of DEC2 in memory formation, we examined whether siRNA-mediated downregulation of DEC2 facilitates spatial learning and memory performance. Mice were randomly divided into two groups and injected in their CA1 area with control siRNA or *DEC2* siRNA. Two days later, they were subjected to water maze learning for two consecutive days, with four trials per day. The retention test started the next day after the end of spatial learning. This shortened schedule was adopted because the effect of *DEC2* siRNA transfection is not long-lasting. Results revealed that mice receiving *DEC2* siRNA transfection showed enhanced acquisition performance compared with mice receiving control siRNA transfection (Fig. S3A). *DEC2* siRNA-transfected mice also spent more time in the target quadrant (Fig. S3B) and traveled more in the target quadrant (Fig. S3C) in the retention test, but swim speed was similar between these two groups of mice (Fig. S3D). Animals were sacrificed after the retention test and their CA1 tissue was dissected out and subjected to Western blot determination of DEC2 expression. Results revealed that *DEC2* siRNA transfection markedly decreased DEC2 expression level in the hippocampus (Fig. S3E).

Conversely, we expect that overexpression of DEC2 would impair spatial learning and memory. To test this, we randomly divided mice into two groups receiving Flag-vector or Flag-DEC2WT plasmid transfection, and then subjected them to water maze learning 48 h later. Results revealed that Flag-DEC2WT plasmid-transfected mice showed impaired acquisition performance compared with Flag-vector-transfected mice (Fig. S3F), spent less time in the target quadrant (Fig. S3G), and traveled less in the target quadrant (Fig. S3H) in the retention test. The swim speed of these two groups of mice was similar (Fig. S3I). Animals were sacrificed after the retention test and their hippocampal tissue was subjected to Western blot determination of DEC2 expression. Results showed that DEC2 expression level was significantly higher in Flag-DEC2WT-transfected mice. Further co-IP experiments confirmed the transfection and expression of DEC2 in Flag-DEC2WT-transfected mice (Fig. S3J).

NCoR1 is suggested to act as a corepressor for transcription activators or transcription repressors, and our current results indicate that NCoR1 is associated with DEC2. It is possible that NCoR1 also functions as a corepressor for DEC2 and that NCoR1 and DEC2 co-regulate gene expression. Because we found that DEC2 suppressed C/EBPα binding to the *Bdnf* promoter and BDNF expression, it is expected that knockdown of NCoR1, which consequently decreases the association between NCoR1 and DEC2, would reduce the suppressive effect of DEC2 on BDNF expression. This issue was examined here by applying ChIP PCR assays in *NCoR1 loxP* mice and *NCoR1* cKO mice. Results revealed that C/EBPα binding to the *Bdnf* promoter was markedly increased in the hippocampus of *NCoR1* cKO mice compared with *NCoR1 loxP* mice (Fig. 3E). Further results of Western blotting experiments indicated that BDNF expression level was significantly increased in the hippocampus of *NCoR1* cKO mice compared with *NCoR1 loxP* mice; conversely, NCoR1 expression level was markedly decreased in *NCoR1* cKO mice compared with *NCoR1 loxP* mice (Fig. 3F).

**Fig. 4:**
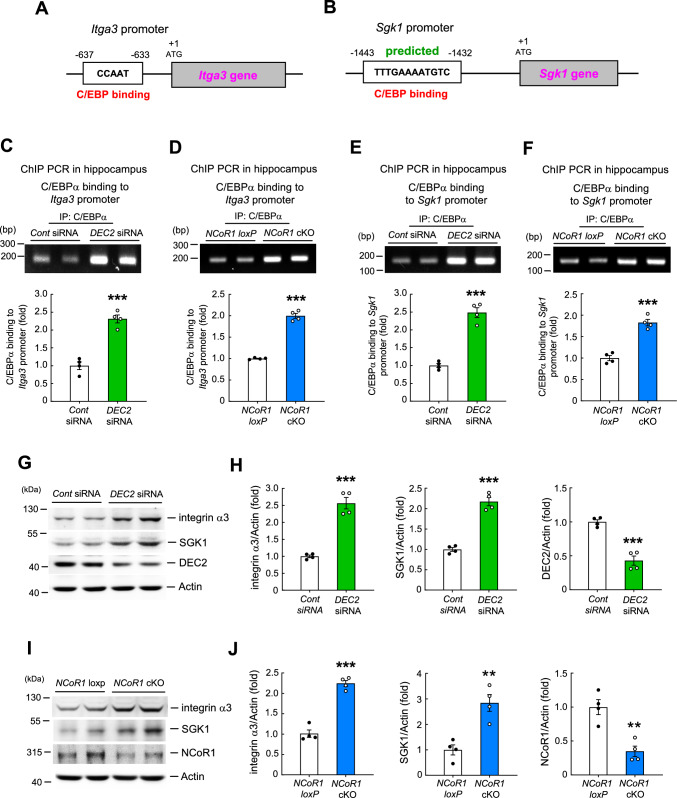
Integrin α3 and SGK1 expression levels are increased in *DEC2* siRNA-transfected mice and in *NCoR1* cKO mice. **A** Promoter analysis shows that the mouse *integrin α3* DNA promoter contains the C/EBP binding element. **B** Promoter analysis prediction shows that the mouse *sgk1* DNA promoter contains the C/EBP binding element. **C** ChIP PCR assay showing C/EBPα binding to the *integrin α3* promoter in the mouse hippocampus from control siRNA- and *DEC2* siRNA (15 pmol)-transfected mice (t_1,6_ = 8.44, *p* < 0.001). **D** ChIP PCR assay showing C/EBPα binding to the *integrin α3* promoter in the mouse hippocampus of *NCoR1 loxP* mice and *NCoR1* cKO mice (t_1,6_ = 17.04, *p* < 0.001). **E** ChIP PCR assay showing C/EBPα binding to the *sgk1* promoter in the hippocampus from control siRNA- and *DEC2* siRNA (15 pmol)-transfected mice (t_1,6_ = 10.06, *p* < 0.001). **F** ChIP PCR assay showing C/EBPα binding to the *sgk1* promoter in the mouse hippocampus of *NCoR1 loxP* mice and *NCoR1* cKO mice (t_1,6_ = 8.57, *p* < 0.001). N = 4 each group for (C) to (F). **G** The expression levels of integrin α3, SGK1 and DEC2 in the hippocampus of control siRNA- and *DEC2* siRNA-transfected mice. **H** The quantified results of (G) (t_1,6_ = 8.93, *p* < 0.001 for integrin α3; t_1,6_ = 10.48, *p* < 0.001 for SGK1 and t_1,6_ = 3.83, *p* < 0.001 for DEC2). N = 4 each group. **I** The expression levels of integrin α3, SGK1 and NCoR1 in the hippocampus of *NCoR1 loxP* mice and *NCoR1* cKO mice. **J** The quantified results of (I) (t_1,6_ = 10.46, *p* < 0.001 for integrin α3; t_1,6_ = 4.84, *p* < 0.01 for SGK1 and t_1,6_ = 4.85, *p* < 0.01 for NCoR1). N = 4 each group. IP: immunoprecipitation. Data are expressed as individual values and mean ± SEM. ** *p* < 0.01 and *** *p* < 0.001

In addition to the *Bdnf* gene, we also examined other genes that are known to facilitate learning and memory and are likely to be negatively regulated by NCoR1. For example, integrin was shown to enhance memory performance in both *Drosophila* and mice [36, 37], and promoter analyses, performed using the same online tools as described above, indicate that the mouse integrin α3 (*Itga3*) gene contains a C/EBP binding element (Fig. 4A). Similarly, SGK1 was found to facilitate spatial learning and memory in rats [10], and the same promoter analysis predicts that the mouse *Sgk1* gene also contains a C/EBP binding element (Fig. 4B). Based on these results and analyses, it is expected that the expression of integrin α3 and SGK1 should be negatively regulated by DEC2 through DEC2 suppression of C/EBPα binding to their DNA promoters. It could be possible that integrin α3 and SGK1 expression is also negatively regulated by NCoR1. To address these possibilities, we divided mice into two groups receiving control siRNA or *DEC2* siRNA (15 pmol) in their CA1 area via transfection. Mice were sacrificed 2 d later, and their hippocampal tissue containing the CA1 area was dissected out and subjected to ChIP PCR assays for determination of C/EBPα binding to the *Itga3* promoter and *Sgk1* promoter. Results revealed that *DEC2* siRNA transfection significantly increased C/EBPα binding to both the *Itga3* promoter (Fig. 4C) and *Sgk1* promoter (Fig. 4E) compared with control siRNA transfection. In a separate experiment, hippocampal tissue lysates from *NCoR1 loxP* mice and *NCoR1* cKO mice were subjected to the same ChIP PCR assays. Results indicated that C/EBPα binding to both the *Itga3* promoter (Fig. 4D) and *Sgk1* promoter (Fig. 4F) was significantly higher in *NCoR1* cKO mice compared with *NCoR1 loxP* mice. Next, we examined whether *DEC2* siRNA transfection similarly increased integrin α3 and SGK1 expression in the same tissue lysates. Results of Western blot analyses indicated that expression levels of integrin α3 and SGK1 were both significantly increased in the hippocampus of *DEC2* siRNA-transfected mice compared with control siRNA-transfected mice. As expected, *DEC2* siRNA markedly decreased the expression level of DEC2 (Fig. 4G and H). Lastly, we examined whether integrin α3 expression and SGK1 expression are similarly increased in *NCoR1* cKO mice compared with *NCoR1 loxP* mice in the same tissue lysates. Western blot analyses revealed that expression levels of both integrin α3 and SGK1 were significantly higher in the hippocampus of *NCoR1* cKO mice compared with *NCoR1 loxP* mice. As expected, NCoR1 expression level was markedly decreased in *NCoR1* cKO mice compared with NCoR1 *loxP* mice (Fig. 4I and J).

### DEC2 and NCoR1 negatively control C/EBPα activity on the promoters of *Bdnf*, *Itga3* and *Sgk1* genes

**Fig. 5:**
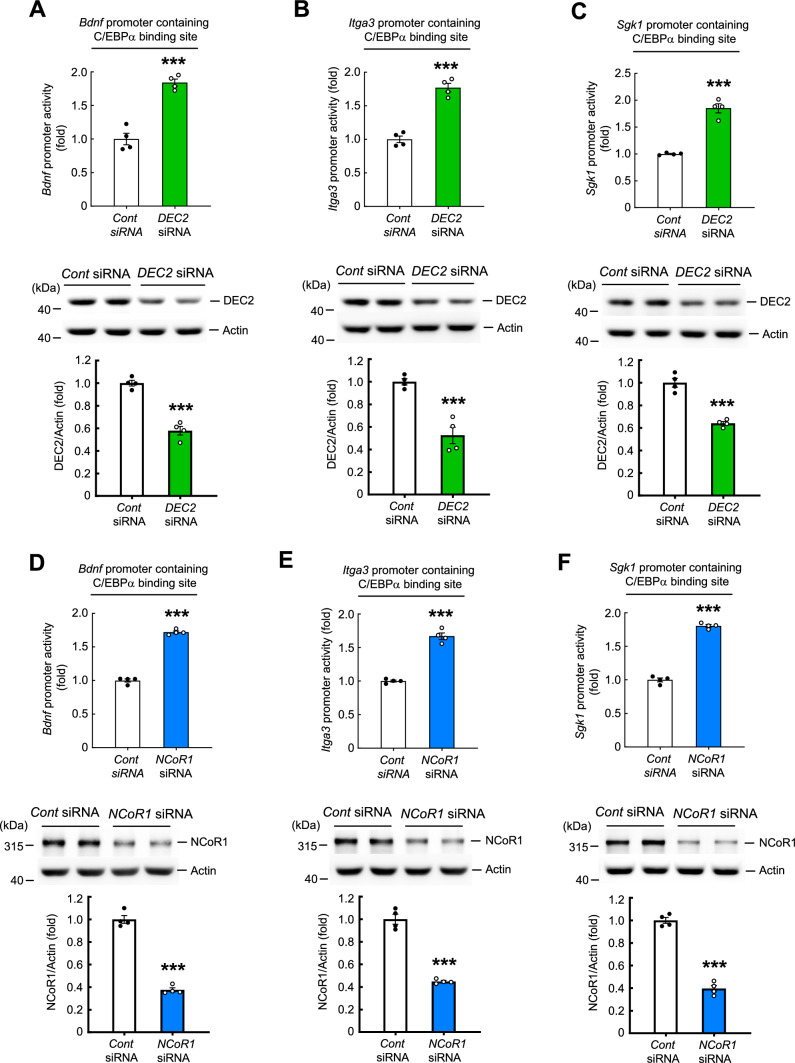
DEC2 and NCoR1 negatively control C/EBPα activity on the promoter of the *bdnf*, *integrin α3* and *sgk1* genes. Control siRNA or *DEC2* siRNA was co-transfected with the *bdnf* promoter containing the C/EBPα binding site, *integrin α3* promoter containing the C/EBPα binding site or *sgk1* promoter containing the C/EBPα binding site to Neuro2A cells and **A** *bdnf* promoter activity (t_1,6_ = 8.34, *p* < 0.001), **B** *integrin α3* promoter activity (t_1,6_ = 9.49, *p* < 0.001) and **C** *sgk1* promoter activity (t_1,6_ = 10.14, *p* < 0.001) was determined by luciferase reporter assay. DEC2 expression level was also determined in these separate experiments by Western blot (t_1,6_ = 9.2, *p* < 0.001; t_1,6_ = 6.07, *p* < 0.001 and t_1,6_ = 8.36, *p* < 0.001, respectively) (**A**–**C**, lower panels). Control siRNA or *NCoR1* siRNA was co-transfected with the *bdnf* promoter containing the C/EBPα binding site, *integrin α3* promoter containing the C/EBPα binding site or *sgk1* promoter containing the C/EBPα binding site to Neuro2A cells and **D** *bdnf* promoter activity (t_1,6_ = 23.18, *p* < 0.001), **E** *integrin α3* promoter activity (t_1,6_ = 13.05, *p* < 0.001) and **F** *sgk1* promoter activity (t_1,6_ = 23.1, *p* < 0.001) was determined by luciferase reporter assay. NCoR1 expression level was also determined in these separate experiments (t_1,6_ = 15.94, *p* < 0.001; t_1,6_ = 12.23, *p* < 0.001 and t_1,6_ = 15.58, *p* < 0.001, respectively) (D-F, lower panels). *** *p* < 0.001

The above results indicate that both DEC2 and NCoR1 negatively regulate C/EBPα binding to *Bdnf*, *Itga3* and *Sgk1* promoters and expression of protein from these genes. In this series of experiments, we further examined whether DEC2 and NCoR1 indeed control C/EBPα activity on the promoters of these three genes employing reporter assays. We first transfected the pcDNA3-EGFP plasmid into mouse primary cultured neurons and evaluated the transfection efficiency. As shown in Fig. S4A and S4B, and consistent with a previous literature report [38], transfection efficiency was very low (~ 2.5%) in primary neurons, making effective assessment of the effect of DEC2 and NCoR1 on gene promoter activity problematic. As an alternative, we evaluated transfection efficiency in Neuro2A cells. Similarly transfecting pcDNA3-EGFP plasmid into Neuro2A cells yielded a transfection efficiency of approximately 16% (Fig. S4C and D), a result consistent with the literature [38]. Therefore, we used Neuro2A cells for all reporter assay experiments. We first examined the effect of DEC2 on C/EBPα activity at the promoters of *Bdnf*, *Itga3,* and *Sgk1* genes. Neuro2A cells were co-transfected with *DEC2* siRNA and the *Bdnf* promoter containing the C/EBPα binding site, the *Itga3* promoter containing the C/EBPα binding site, or the *Sgk1* promoter containing the C/EBPα binding site, and luciferase assays were performed. Results indicated that *DEC2* siRNA transfection significantly increased activity of *Bdnf*, *Itga3f*, and *Sgk1* promoters (Fig. 5A–C, upper panels). As expected, *DEC2* siRNA transfection markedly decreased expression levels of DEC2 in each of these experiments (Fig. 5A–C, lower panels). Next, we examined the effect of NCoR1 on C/EBPα activity at the promoters of *Bdnf*, *Itga3,* and *Sgk1* genes. *NCoR1* siRNA was co-transfected into Neuro2A cells together with C/EBPα binding site-containing *Bdnf*, *Itga3,* or *Sgk1* promoter, and luciferase assays were conducted. Results indicated that *NCoR1* siRNA transfection significantly increased the activity of *Bdnf*, *Itga3* and *Sgk1* promoters (Fig. 5D–F, upper panels). In addition, *NCoR1* siRNA transfection decreased expression levels of NCoR1 in each of these experiments (Fig. 5D–F, lower panels).

### C/EBPα binding to *Bdnf*, *Itga3*, and *Sgk1* promoter is increased in fast learners and trained animals in conjunction with increases in BDNF, integrin α3 and SGK1 protein expression

**Fig. 6:**
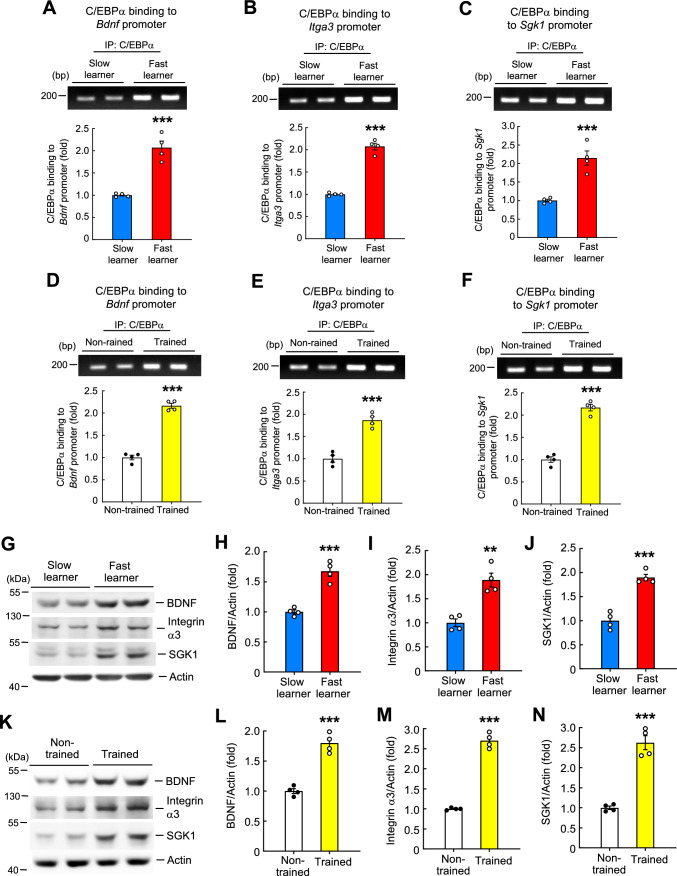
C/EBPα binding to the *bdnf* promoter, *integrin α3* promoter and *sgk1* promoter as well as BDNF, integrin α3 and SGK1 protein expression are increased in fast learners and in trained animals. **A** ChIP PCR assay showing C/EBPα binding to the *bdnf* promoter in the hippocampus of fast learners and slow learners (t_1,6_ = 7.19, *p* < 0.001). **B** ChIP PCR assay showing C/EBPα binding to the *integrin α3* promoter in the hippocampus of fast learners and slow learners (t_1,6_ = 13.67, *p* < 0.001). **C** ChIP PCR assay showing C/EBPα binding to the *sgk1* promoter in the hippocampus of fast learners and slow learners (t_1,6_ = 5.87, *p* = 0.001). **D** ChIP PCR assay showing C/EBPα binding to the *bdnf* promoter in the hippocampus of non-trained and trained animals (t_1,6_ = 15.25, *p* < 0.001). **E** ChIP PCR assay showing C/EBPα binding to the *integrin α3* promoter in the hippocampus of non-trained and trained animals (t_1,6_ = 7.76, *p* < 0.001). **F** ChIP PCR assay showing C/EBPα binding to the *sgk1* promoter in the hippocampus of non-trained and trained animals (t_1,6_ = 12.05, *p* < 0.001). The same tissue lysates from Fig. 1 were subjected to Western blot analysis of BDNF, integrin α3 and SGK1 expression. **G** Representative gel pattern of these protein expressions in slow learners and fast learners. **H** BDNF expression level in slow learners and fast learners (t_1,6_ = 7.56, *p* < 0.001). **I** Integrin α3 expression level in slow learners and fast learners (t_1,6_ = 5.45, *p* < 0.01). **J** SGK1 expression level in slow learners and fast learners (t_1,6_ = 8.15, *p* < 0.001). **K** Representative gel pattern of these protein expressions in non-trained and trained animals. **L** BDNF expression level in non-trained and trained animals (t_1,6_ = 8.86, *p* < 0.001). **M** Integrin α3 expression level in non-trained and trained animals (t_1,6_ = 18.2, *p* < 0.001). **N** SGK1 expression level in non-trained and trained animals (t_1,6_ = 9.07, *p* < 0.001). N = 4 each group. *IP* immunoprecipitation. Data are expressed as individual values and mean ± SEM. *** *p* ≤ 0.001

As shown in Fig. 1, NCoR1 expression levels are lower in fast learners than slow learners, and are also lower in trained animals compared with non-trained controls. The above results further showed that C/EBPα binding to *Bdnf*, *Itga3* and *Sgk1* promoters is increased in *NCoR1* cKO mice compared with *NCoR1 loxP* mice. Based on these findings, we expect that C/EBPα binding to *Bdnf*, *Itga3*, and *Sgk1* promoters should be higher in fast learners than slow learners and in trained animals compared with non-trained animals. ChIP PCR assays designed to test these predictions showed that C/EBPα binding to *Bdnf*, *Itga3* and *Sgk1* promoters was significantly higher in the hippocampus of fast learners than slow learners (Fig. 6A–C). They further showed that C/EBPα binding to *Bdnf*, *Itga3*, and *Sgk1* promoters was markedly higher in the hippocampus of trained animals compared with non-trained animals (Fig. 6D–F).

Having established that C/EBPα binding to *Bdnf*, *Iga3* and *Sgk1* promoters is increased in fast learners and trained animals, we next examined whether BDNF, integrin α3 and SGK1 protein expression are also increased in the same animals. Western blot analyses revealed that expression levels of BDNF, integrin α3, and SGK1 were markedly higher in the hippocampus of fast learners than slow learners (Fig. 6G–J). Expression levels of BDNF, integrin α3 and SGK1 were also significantly higher in the hippocampus of trained animals compared with non-trained animals (Fig. 6K–N).

### Overexpression of DEC2 in *NCoR1* cKO mice rescues the decreased expression of BDNF, integrin α3 and SGK1 compared with *NCoR1 loxP* mice overexpressed with DEC2

**Fig. 7:**
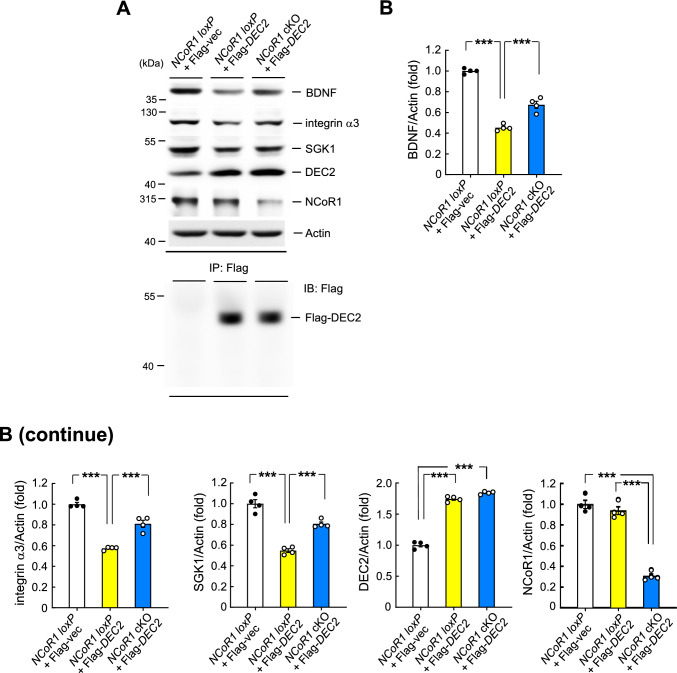
Overexpression of DEC2 in *NCoR1* cKO mice rescues the decreased expression of BDNF, integrin α3 and SGK1 compared with *NCoR1 loxP* mice overexpressed with DEC2. **A** Flag-vector or Flag-*DEC2* plasmid was transfected to the CA1 area of *NCoR1 loxP* mice, and Flag-*DEC2* plasmid was transfected to the CA1 area of *NCoR1* cKO mice. Their hippocampal tissue was dissected out 48 h later and subjected to Western blot determination of BDNF, integrin α3, SGK1, DEC2 and NCoR1 expressions. Plasmid transfection and expression was confirmed by IP: Flag and IB: Flag (lower panel). **B** The quantified results of these protein expressions (F_2,9_ = 144.92, *p* < 0.001 for BDNF; q = 23.93, *p* < 0.001 comparing the *NCoR1 loxP* + Flag-*DEC2* group with *NCoR1 loxP* + Flag-vector group; q = 9.66, *p* < 0.001 comparing the *NCoR1* cKO + Flag-*DEC2* group with *NCoR1 loxP* + Flag-*DEC2* group) (F_2,9_ = 86.22, *p* < 0.001 for integrin α3; q = 18.53, *p* < 0.001 comparing the *NCoR1 loxP* + Flag-*DEC2* group with *NCoR1 loxP* + Flag-vector group; q = 10.29, *p* < 0.001 comparing the *NCoR1* cKO + Flag-*DEC2* group with *NCoR1 loxP* + Flag-*DEC2* group) (F_2,9_ = 67.96, *p* < 0.001 for SGK1; q = 16.43, *p* < 0.001 comparing the *NCoR1 loxP* + Flag-*DEC2* group with *NCoR1 loxP* + Flag-vector group; q = 9.37, *p* < 0.001 comparing the *NCoR1* cKO + Flag-*DEC2* group with *NCoR1 loxP* + Flag-*DEC2* group) (F_2,9_ = 460.84, *p* < 0.001 for DEC2; q = 34.62, *p* < 0.001 comparing the *NCoR1 loxP* + Flag-*DEC2* group with *NCoR1 loxP* + Flag-vector group; q = 39.3, *p* < 0.001 comparing the *NCoR1* cKO + Flag-*DEC2* group with *NCoR1 loxP* + Flag-vector group) (F_2,9_ = 140.28, *p* < 0.001 for NCoR1; q = 19.51, *p* < 0.001 comparing the *NCoR1* cKO + Flag-*DEC2* group with *NCoR1 loxP* + Flag-*DEC2* group; q = 21.39, *p* < 0.001 comparing the *NCoR1* cKO + Flag-*DEC2* group with *NCoR1 loxP* + Flag-vector group). N = 4 each group. Data are expressed as individual values and mean ± SEM. *** *p* < 0.001

The above results revealed that both DEC2 and NCoR1 negatively regulate the C/EBPα-mediated expression of BDNF, integrin α3 and SGK1, and that spatial training is associated with decreased interaction between DEC2 and NCoR1. However, these results do not demonstrate that NCoR1 acts through DEC2 to downregulate BDNF, integrin α3 and SGK1 expression. The ideal approach for examining this issue would be to knockdown DEC2 in NCoR1-overexpressing mice and examine whether this manipulation alters the expression of BDNF, integrin α3 and SGK1, or to overexpress NCoR1 in *NCoR1* cKO mice and examine the interaction between NCoR1 and DEC2 and the regulation of BDNF, integrin α3 and SGK1. However, because the molecular weight of the NCoR1 protein (> 300 kDa) makes it difficult to ligate sequences encoding NCoR1 into the lentiviral vector and successfully express NCoR1 in the mouse hippocampus, we addressed this issue using an alternative approach. Three groups of mice were used for this experiment. In the first group, the Flag-vector plasmid was transfected into the CA1 area of *NCoR1 loxP* mice (control group); in the second group, the Flag-*DEC2* plasmid was transfected into the CA1 area of *NCoR1 loxP* mice; and in the third group, the Flag-*DEC2* plasmid was transfected into the CA1 area of *NCoR1* cKO mice. Animals were sacrificed 48 h after plasmid transfection and their hippocampal tissue containing the CA1 area was subjected to Western blot determination of BDNF, integrin α3 and SGK1 expression. Results revealed that transfection of Flag-*DEC2* plasmid into *NCoR1 loxP* mice significantly decreased the expression of BDNF, integrin α3 and SGK1 compared with that in *NCoR1 loxP* mice transfected with Flag-vector. However, Flag-*DEC2* plasmid transfection into *NCoR1* cKO mice partially, but significantly, rescued the decreased expression of these proteins compared with Flag-*DEC2* plasmid transfection into *NCoR1 loxP* mice (Fig. 7A and B). Immunoprecipitating the same tissue lysates with anti-Flag antibody followed by immunoblotting with anti-Flag antibody confirmed the transfection and expression of DEC2 in the hippocampus (Fig. 7A, lower panel).

### The JNK signaling pathway regulates NCoR1 expression, and JNK activity is decreased in fast learners and trained animals

**Fig. 8:**
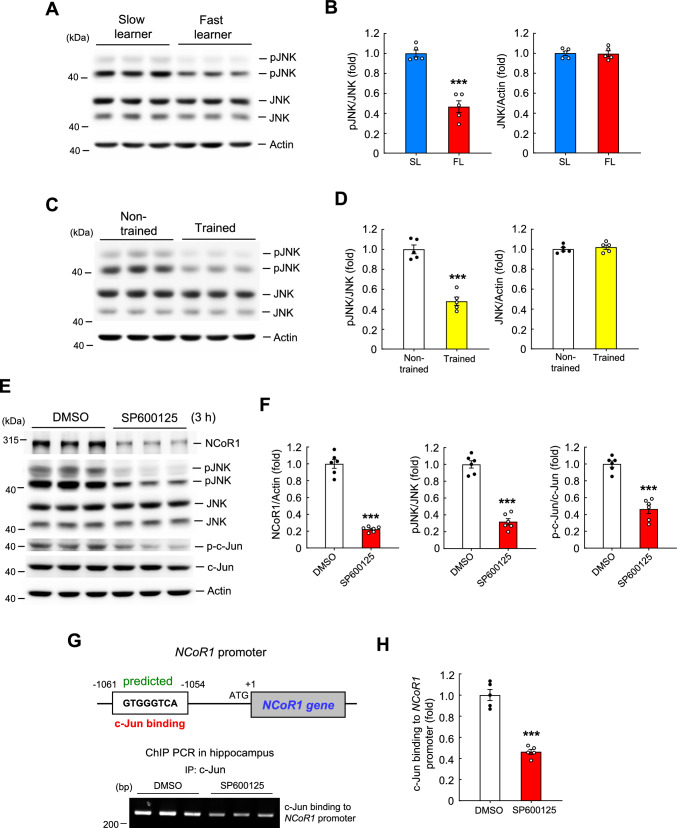
JNK activation is decreased in fast learners and in trained animals and NCoR1 expression is regulated by the JNK signaling pathway. **A** JNK phosphorylation level (pJNK) and JNK expression level in the CA1 area of slow learners and fast learners. **B** The quantified result of **A** (t_1,8_ = 7.85, *p* < 0.001 for pJNK/JNK and t_1,8_ = 0.11, *p* > 0.05 for JNK/Actin). **C** JNK phosphorylation level (pJNK) and JNK expression level in the CA1 area from non-trained and trained rats. **D** The quantified result of **C** (t_1,8_ = 8.57, *p* < 0.001 for pJNK/JNK and t_1,8_ = 0.71, *p* > 0.05 for JNK/Actin). **E** Mice received acute DMSO (45%) or SP600125 (4.5 mM) administration to their hippocampal CA1 area and their CA1 tissue was dissected out 3 h later for various Western blot assays. N = 6 each group. **F** The quantified results of (E) for NCoR1 expression (t_1,10_ = 13.6, *p* < 0.001), pJNK/JNK expression (t_1,10_ = 11.82, *p* < 0.001) and p–c-Jun/c-Jun expression (t_1,10_ = 8.6, *p* < 0.001). **G** Promoter analysis prediction shows that the mouse *NCoR1* DNA promoter contains the c-Jun binding element (AP-1 site) (upper panel). ChIP PCR assay showing c-Jun binding to the *NCoR1* promoter in the mouse hippocampus from DMSO (45%)- and SP600125 (4.5 mM)-treated CA1 tissues (lower panel). IP: immunoprecipitation.** H** The quantified result of **G** from five independent experiments (t_1,8_ = 9.27, *p* < 0.001). Data are expressed as individual values and mean ± SEM. *** *p* < 0.001

Our results showed that NCoR1 expression levels are lower in fast learners than slow learners and that spatial training decreases the expression of NCoR1. Here, we examined which signaling pathway regulates spatial learning-associated expression of NCoR1. Protein kinases are suggested to be involved in memory formation [39, 40]. One such protein kinase is c-Jun N-terminal kinase (JNK), whose activation has been shown to negatively regulate synaptic plasticity and spatial memory [41]. Thus, we first examined whether JNK activation levels are different between fast learners and slow learners. We found that levels of phosphorylated (activated) JNK were lower in fast learners than slow learners, but overall JNK expression levels were similar between these two groups of mice (Fig. 8A and B). Similar results were obtained with spatial training. Specifically, results indicated that levels of phosphorylated JNK were decreased in trained animals compared with non-trained animals, with no change in total JNK expression level (Fig. 8C and D). Next, we examined whether JNK signaling regulates NCoR1 expression. Mice were randomly divided to two groups receiving an injection of either vehicle (45% DMSO) or the JNK inhibitor SP600125 (4.5 mM) into their CA1 area. Their hippocampal tissue was dissected out and subjected to Western blot analyses 3 h later. Results showed that acute SP600125 administration significantly decreased NCoR1 expression. It also dramatically decreased the levels of phosphorylated JNK without altering total JNK expression levels, confirming the effectiveness of SP600125 administration (Fig. 8E and F). We further examined the levels of phosphorylated and total c-Jun, a downstream target of JNK signaling. Results indicated that SP600125 consistently decreased the level of phosphorylated c-Jun without changing total c-Jun expression level (Fig. 8E and F).

The above results suggest that JNK/c-Jun signaling regulates NCoR1 expression. A promoter analysis, performed as described above, predicts that the mouse *NCoR1* gene contains an AP-1 site, which is specific for c-Jun DNA binding (Fig. 8G, upper panel), suggesting that *NCoR1* gene expression is directly regulated by c-Jun. To test this, we conducted ChIP PCR assays to determine c-Jun binding to the *NCoR1* promoter in DMSO-injected and SP600125-injected mouse hippocampal tissues. Results showed that SP600125 significantly decreased c-Jun binding to the *NCoR1* promoter compared with DMSO administration (Fig. 8G, lower panel and Fig. 8H).

### Spatial learning and memory is impaired by siRNA-mediated knockdown of BDNF, integrin α3 or SGK1, but is facilitated by inhibition of JNK with SP600125

**Fig. 9:**
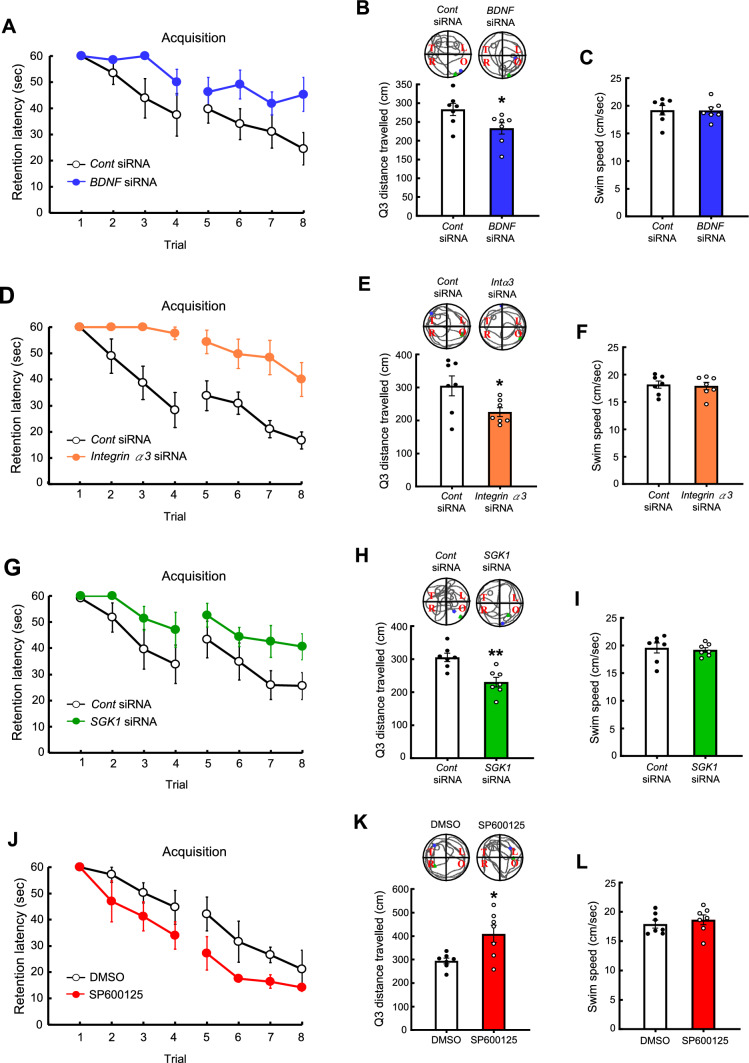
*BDNF* siRNA, *integrinα3* siRNA and *SGK1* siRNA transfections impair spatial learning and memory, but SP600125 facilitates spatial learning and memory. **A** Mice were divided to two groups and received control siRNA or *BDNF* siRNA transfection to their hippocampus and were subjected to water maze learning 48 h later. They were subjected to the probe trial test one day later and **B** the distance travelled in the target quadrant and the representative swim patterns, and **C** swim speed are shown. **D** A different batch of mice were divided to two groups and received control siRNA or *integrin a3* siRNA transfection to their hippocampus and were subjected to water maze learning 48 h later. They were subjected to the probe trial test one day later and **E** the distance travelled in the target quadrant and the representative swim patterns, and **F** swim speed are shown. **G** A separate group of mice were divided to two groups and received control siRNA or *SGK1* siRNA transfection to their hippocampus and were subjected to water maze learning 48 h later. They were subjected to the probe trial test one day later and **H** the distance travelled in the target quadrant and the representative swim patterns, and **I** swim speed are shown. **J** A different group of mice were divided to two groups and received DMSO (45%) or SP600125 (4.5 mM) infusion to their hippocampus and were subjected to water maze learning 48 h later. They were subjected to the probe trial test one day later and **K** the distance travelled in the target quadrant and the representative swim patterns, and **L** swim speed are shown. N = 7 each group. Data are expressed as mean ± SEM or individual values and mean ± SEM. * *p* < 0.05 and ** *p* < 0.01

Results above indicate that DEC2 and NCoR1 negatively regulate the expression of BDNF, integrin α3 and SGK1 through C/EBPα, and that BDNF, integrin α3 and SGK1 expression levels are higher in fast learners than slow learners and in trained animals compared with non-trained controls. Here, we examined whether knockdown of these proteins impairs spatial learning and memory. In the first experiment, control siRNA or *BDNF* siRNA was transfected into the mouse hippocampus and water maze learning—four trials per day for two consecutive days—was carried out 48 h later. Results showed that *BDNF* siRNA significantly impaired spatial acquisition (Fig. 9A). It also decreased the distance that animals traveled in the target quadrant during the probe trial test (Fig. 9B), but the swim speed was similar between these two groups of mice (Fig. 9C). In the next experiment, control siRNA or *integrin α3* siRNA was similarly transfected into the mouse hippocampus and spatial learning and memory was tested 48 h later using the same paradigm. Results revealed that integrin α3 knockdown markedly impaired acquisition performance (Fig. 9D). It also reduced the distance that animals traveled in the target quadrant during the probe trial test (Fig. 9E) without affecting the swim speed (Fig. 9F). Next, we assessed the effect of knocking down SGK1 expression on spatial learning and memory performance. Control siRNA or *SGK1* siRNA was transfected into the mouse hippocampus and water maze learning tests were conducted 48 h later using the same paradigm. Results indicated that *SGK1* siRNA significantly impaired spatial acquisition (Fig. 9G). It also decreased the distance that animals traveled in the target quadrant for the probe trial test (Fig. 9H); again, the swim speed of control siRNA- and *SGK1* siRNA-transfected animals was similar (Fig. 9I). We showed above that SP600125 decreases NCoR1 expression through inhibition of JNK activation, and that both fast learners and trained animals have lower levels of activated (phosphorylated) JNK, but whether inhibition of JNK with SP600125 indeed facilitates spatial learning and memory performance is not known. In our final experiment, we examined this issue by infusing DMSO or SP600125 into the mouse hippocampus and conducting spatial learning and memory tests 48 h later. Results showed that SP600125 administration markedly enhanced acquisition performance (Fig. 9J). It also increased the distance that animals traveled in the target quadrant during the probe trial test (Fig. 9K) without affecting swim speed (Fig. 9L).

### siRNA-mediated knockdown of BDNF, integrin α3, or SGK1 does not affect Y-maze performance in mice

**Fig. 10:**
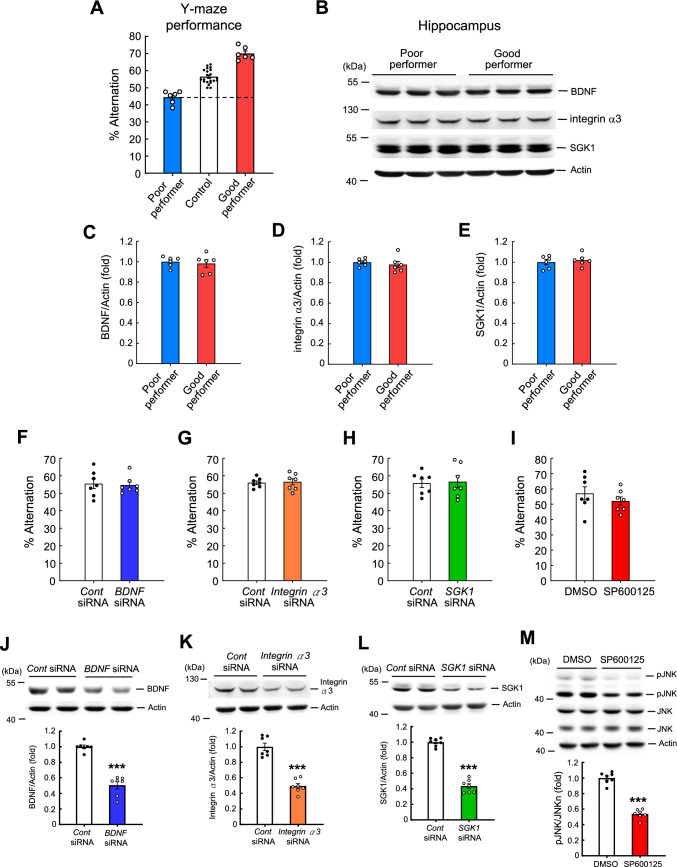
*BDNF* siRNA, *integrinα3* siRNA and *SGK1* siRNA transfections do not affect Y-maze performance in mice. **A** Thirty three naive mice were subjected to the Y-maze test and their spontaneous alternation performance is shown (N = 6 each for the poor performer and good performer groups and N = 21 for the control group). **B** A representative gel pattern showing BDNF, integrin α3 and SGK1 expression levels of the poor performers and good performers. Quantified results for **C** BDNF expression (t_1,10_ = 0.41, *p* > 0.05), **D** integrin α3 expression (t_1,10_ = 0.62, *p* > 0.05) and **E** SGK1 expression (t_1,10_ = 0.56, *p* > 0.05) in these animals (N = 6 each group). Animals used for the water maze experiment from Fig. 9 were also subjected to the Y-maze test 5 h after the probe trial test. The spontaneous alternation performance of animals transfected with **F** control siRNA and *BDNF* siRNA (t_1,12_ = 0.23, *p* > 0.05) **G** control siRNA and *integrin α3* siRNA (t_1,12_ = 0.24, *p* > 0.05) and **H** control siRNA and *SGK1* siRNA (t_1,12_ = 0.21, *p* > 0.05) is shown. **I** Spontaneous alternation performance of mice treated with DMSO and SP600125 is shown (t_1,12_ = 0.99, *p* > 0.05). Animals were sacrificed after the Y-maze test and their hippocampal tissue was subjected to Western blot analysis of **J** BDNF expression (t_1,12_ = 9.53, *p* < 0.001) **K** integrin α3 expression (t_1,12_ = 8.7, *p* < 0.001) **L** SGK1 expression (t_1,12_ = 15.09, *p* < 0.001) and **M** pJNK expression (t_1,12_ = 12.97, *p* < 0.001 for pJNK/JNK and t_1,12_ = 1.99, *p* > 0.05 for JNK/Actin). N = 7 each group for 10F-10 M. Data are expressed as individual values and mean ± SEM. *** *p* < 0.001

The above results collectively indicate that BDNF, integrin α3, and SGK1 are differentially expressed between slow learners and fast learners as well as between non-trained and trained animals in the water maze learning task. Although previous studies have shown that integrin α is involved in short-term olfactory memory in *Drosophila* [36, 42], whether the expression of BDNF, integrin α3, and/or SGK1 proteins is also altered in short-term memory in mice is not known. To examine whether BDNF, integrin α3 and SGK1 are involved in short-term memory, we applied the Y-maze task, in which spontaneous alternations provide a measure of hippocampal function-dependent short-term spatial working memory [24, 25]. In the first experiment, we chose good performers (i.e., those with spontaneous alternations > 65%) and poor performers (with spontaneous alternations < 48%) from among the 33 mice examined. In so doing, we were able to achieve a mean difference in performance greater than 35%. The remaining mice were assigned to the control group (Fig. 10A). We then examined BDNF, integrin α3, and SGK1 expression in good performers and poor performers (Fig. 10B). Results revealed that expression levels of BDNF, integrin α3, and SGK1 were similar between these two groups of mice (Fig. 10C–E). Next, we evaluated the Y-maze performance of the same mice transfected with *BDNF* siRNA, *integrin α3* siRNA or *SGK1* siRNA used in the previous water maze experiment. Results showed that transfection of *BDNF* siRNA, *integrin α3* siRNA or *Sgk1* siRNA does not affect the Y-maze performance of these animals (Fig. 10F–H). Animals were sacrificed after the Y-maze test, and their hippocampal BDNF, integrin α3 and SGK1 expression levels were examined. Results indicated that transfection of siRNA against *BDNF*, *integrin α3* and *SGK1* markedly decreased expression levels of the corresponding protein (Fig. 10J–L). We also examined the effect of SP600125 on Y-maze performance using the same animals previously subjected to water maze learning. The results revealed that SP600125 administration did not affect Y-maze performance (Fig. 10I), despite significantly decreasing the JNK phosphorylation level (Fig. 10M).

## Discussion

**Fig. 11:**
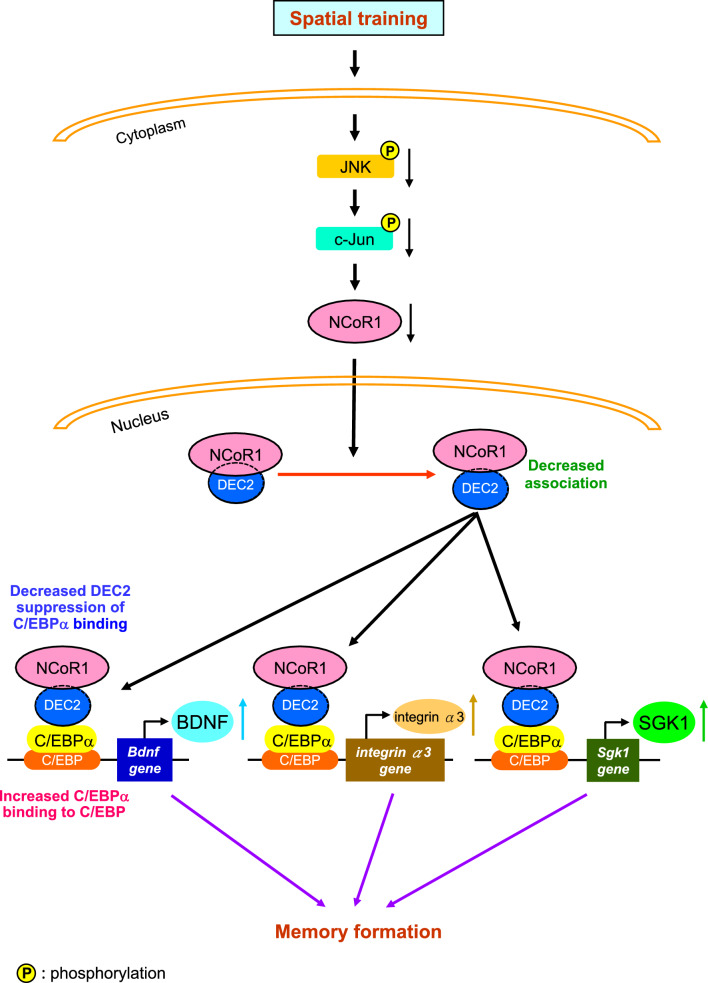
Illustration showing the signaling pathway and molecular mechanism of spatial training-induced downregulation of NCoR1 expression and upregulation of BDNF, integrin α3 and SGK1 expression that leads to memory formation

**Fig. 12:**
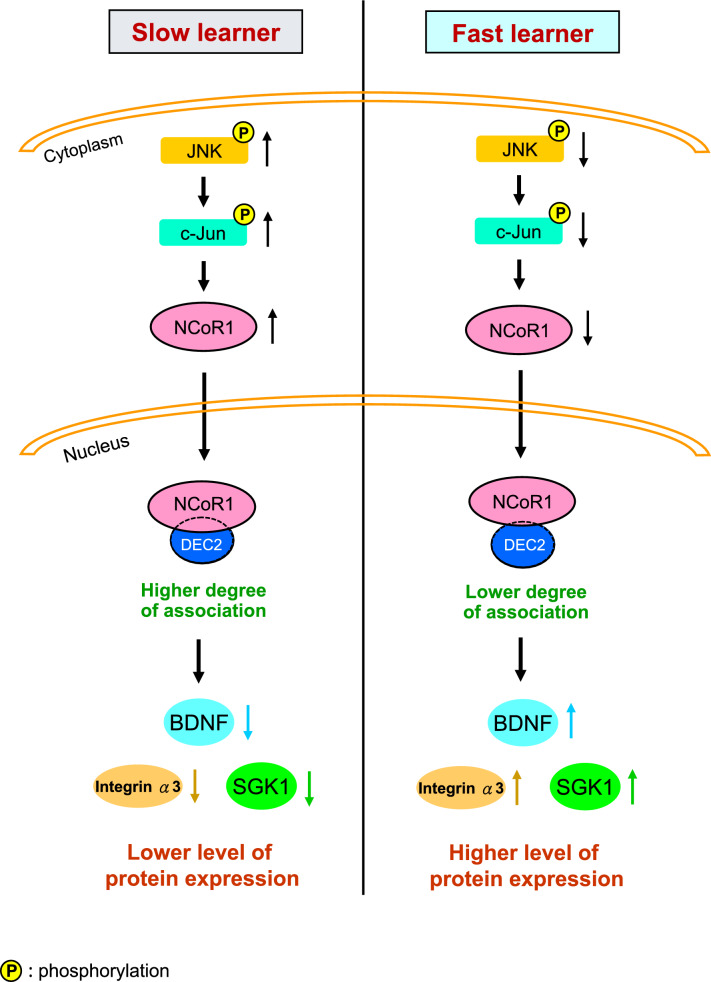
Illustration showing the signaling pathway and molecular mechanism in regulation of NCoR1, BDNF, integrin α3 and SGK1 expression in slow learners and fast learners from the water maze learning task

Through DD-PCR, we here identified the *NCoR1* gene as being negatively associated with spatial memory formation. Our results further indicate that *NCoR1* cKO mice show enhanced acquisition and retention performance in the water maze learning task. One of the mechanisms mediating the memory-impairing effect of NCoR1 is its interaction with DEC2, which acts through C/EBPα to suppress BDNF, integrin α3 and SGK1 expression. Moreover, NCoR1 expression is regulated by the JNK/c-Jun signaling pathway. Spatial training induces downregulation of NCoR1 expression and upregulation of BDNF, integrin α3, and SGK1 expression through the signaling pathway and molecular mechanism summarized in Fig. 11. In addition, endogenous NCoR1 expression levels are higher in slow learners and lower in fast learners. This is attributable to differential activation of the JNK pathway, which affects the interaction between NCoR1 and DEC2 and results in changes in the expression levels of BDNF, integrin α3, and SGK1. These differential signaling pathways and molecular interactions are summarized in Fig. 12.

Our results are consistent with the fact that NCoR1 is a transcriptional corepressor and the general idea that corepressor-regulated gene expression is important for cognitive function [43]. However, another study has shown that depletion of both NCoR1 and NCoR2 impairs memory performance in mice [44]. Our results may not conflict with this previous observation because NCoR1 may play different roles in different brain regions through distinct mechanisms. Specifically, in the latter study, the role of both NCoR1 and NCoR2 was examined specifically in GABAergic neurons, whereas we only examined the role of NCoR1 in the hippocampus. In addition, the previous study examined NCoR regulation of GABA neuronal signaling from the hypothalamus to the CA3 area, whereas we investigated the role of NCoR1 primarily in the CA1 area. In the current study, we did not examine the role of NCoR2 in spatial learning and memory because the *NCoR2* cDNA fragment was not identified in our DD-PCR screens.

DEC2 is a transcriptional repressor that regulates circadian clock and sleep in mammals [29, 45]. In the current study, we found that DEC2 suppresses the expression of BDNF, integrin α3 and SGK1 proteins, which facilitate learning and memory performance, and that knockdown of DEC2 facilitates spatial memory formation in mice. Our results are incongruent with a study reporting that only *Dec1*/*Dec2*-double KO mice, and not *Dec2* KO mice, show enhanced memory consolidation [30]. We are currently unable to explain this discrepancy, but would note that different learning tasks were adopted and different brain regions were examined in these two studies, which may partly account for the different results obtained. Another possibility is a compensatory mechanism in *Dec2* KO mice wherein DEC1, which shares 97% homology with DEC2 in the bHLH region involved in DNA binding [27] (despite showing only 42% homology in overall amino acid sequence with DEC2), may have substituted for DEC2 in *Dec2* KO mice, preventing improved memory performance from being observed in these single knockout mice. Notably, such compensation would not occur in *DEC2* siRNA-transfected mice. In addition to its role in regulation of memory, NCoR1 has also been found to regulate circadian physiology through repression of the expression of the Nr1d1-regulated clock gene, *Bmal1* [46]. Thus, it is possible that NCoR1 interaction with DEC2 may also participate in the regulation of circadian clock gene expression and sleep. On the other hand, the present results do not firmly establish a direct interaction between NCoR1 and DEC2, for which interaction domains have not been previously reported. Although NCoR1 may nonetheless interact directly with DEC2, it is possible that DEC2 is another component of the NCoR1 complex and that NCoR1 indirectly interacts with DEC2 through other proteins in this complex, such as HDAC3. Our results also do not reveal whether NCoR1 and DEC2 regulate spatial memory bi-directionally.

In addition to BDNF, integrin α3 and SGK1 have also been found to play an important role in LTP and memory formation [10, 11, 47, 48]. Although we showed in this study that both NCoR1 and DEC2 negatively regulate the expression of these proteins through the intermediary, C/EBPα, we did not provide evidence that NCoR1 decreases the expression of these proteins through DEC2. This is because of the difficulty of successfully cloning and transducing the large lentiviral NCoR1 vector into the mouse brain before performing the requisite *DEC2* siRNA transfection necessary to examine this issue. But we did address this issue using an alternative approach by overexpressing DEC2 in the hippocampus of *NCoR1* cKO mice, finding that it effectively rescued the decreased expression of BDNF, integrin α3 and SGK1 compared with that observed in DEC2-overexpressing *NCoR1 loxP* mice. Because NCoR1 interacts with DEC2 in the context of spatial learning, and NCoR1 and DEC2 both impair spatial memory, these results collectively suggest that DEC2 is, at least, among the downstream mediators of NCoR1’s negative regulation of the expression of these proteins and spatial memory formation. On the other hand, overexpression of DEC2 in *NCoR1* cKO mice did not completely reverse the decreased expression of BDNF, integrin α3 and SGK1 compared with DEC2-overexpressing *NCoR1 loxP* mice (Fig. 7). This suggests that there may be other molecules in addition to DEC2 that also mediate the effect of NCoR1 in negatively regulating the expression of these proteins and spatial memory formation.

To address the question of how spatial training decreases NCoR1 expression, we examined the role of the JNK/c-Jun signaling pathway. This is because the JNK pathway has been shown to mediate NMDA receptor hyperactivation-induced excitotoxicity [49] and NMDA receptor activation plays an essential role in mediating the effect of learning [31]. Our results show that spatial training decreases the phosphorylation level of JNK, and that fast learners similarly show a lower level of JNK phosphorylation. Conversely, the JNK inhibitor, SP600125, decreases NCoR1 expression. These findings suggest that spatial learning downregulates NCoR1 expression through inhibition of JNK-mediated signaling. This result is also congruent with the finding that JNK inhibits AMPA receptor GluR1 subunit expression [50] because the AMPA receptor plays an important role in synaptic plasticity [51]. c-Jun is a transcription factor that acts through binding to DNA to regulate transcription [52]. Our ChIP assay results showed that c-Jun directly binds to the DNA promoter of the *NCoR1* gene and that inhibition of JNK by SP600125 decreases c-Jun binding to the *NCoR1* promoter. These results are consistent with a report that JNK activation negatively regulates spatial memory [41].

In the present study, the *NCoR1* gene was differentially expressed between fast learners and slow learners. Since fast learners and slow learners were subjected to different numbers of training trials, it is reasonable to suppose that experiencing different numbers of trials and amount of time during the task could affect *NCoR1* mRNA and protein expression. To resolve this issue, we conducted an additional experiment in which naive rats were divided into a trained group and a non-trained group and subjected to the same number of training trials and trial duration, after which their *NCoR1* mRNA and protein expression were examined. The results of this experiment showed that *NCoR1* mRNA and protein expression levels were significantly different between the non-trained rats and trained rats, and the extent of the difference between these two groups of rats (Fig. 1F and G) was similar to that between fast learners and slow learners (Fig. 1D and E). These results suggest that the difference in *NCoR1* mRNA and protein expression between fast learners and slow learners is not due to different trial numbers or duration experienced by the animals in the learning task. In addition, the slow learners eventually learned the location of the hidden platform, but their hippocampal NCoR1 expression level was still higher than that of the fast learners. This is probably because expression of other genes during the acquisition process, such as c-Fos and Zif268, contribute to learning performance.

In the present study, animals in the control group in some water maze experiments performed relatively weakly (~ 30%) in the "time spent in the target quadrant" measure in the probe trial test. This is probably because of the relatively high variability in the control group. Similar results have also been reported in other studies [22, 53, 54]. To confirm our observed effects, we adopted another measure—"distance traveled in the target quadrant"—and found that this measure was consistent with the "time spent in the target quadrant" measure for all experiments conducted.

The present results revealed that BDNF, integrin α3 and SGK1 all facilitate spatial learning and memory, a cognitive task that requires hippocampal function. Because integrin α is demonstrated to be involved in short-term olfactory memory in *Drosophila* [34, 42], we also assessed whether BDNF, integrin α3 and SGK1 are involved in short-term memory in mice by adopting the Y-maze learning task, a task that also requires hippocampal function. Our results showed that the expression levels of these three proteins were not different between poor performers and good performers in the Y-maze task. Knocking down expression of these proteins also did not alter the Y-maze performance of mice. Taken together, these results suggest that BDNF, integrin α3 and SGK1 are involved in long-term memory formation, but not short-term memory formation, in mice.

In this study, we identified a novel role for NCoR1 in the negative regulation of spatial memory formation. Our findings are consistent with the idea that, in addition to memory-enhancing genes, memory-suppressing genes are as important in the establishment of long-term memory [15]. Our results are also congruent with the concept that memory-suppressor genes can modulate different stages of memory processing, including acquisition and consolidation [55]. We also identified DEC2 as a novel NCoR1-interacting protein. NCoR1 interaction with DEC2 negatively regulates spatial memory through suppression of C/EBPα-mediated expression of a few proteins that are known to facilitate memory. Moreover, spatial training inhibits JNK/c-Jun signaling-regulated NCoR1 expression and results in memory facilitation. These results advance our understanding of the molecular mechanism of long-term memory formation in mammals. They may also provide novel therapeutic directions for treating memory disorders through the discovery of drugs that target suppression of NCoR1 expression.

### Supplementary Information

Below is the link to the electronic supplementary material.

Supplementary file1 (PDF 20 kb)

Supplementary file2 (PDF 114 kb)

Supplementary file3 (PDF 190 kb)

Supplementary file4 (PDF 170 kb)

Supplementary file5 (PDF 627 kb)

Supplementary file6 (PDF 3872 kb)

## Data Availability

The data used/analyzed in the present study are available from the corresponding author upon reasonable request. The materials used in the present study are available from the corresponding author.
