# Supplementary material for: Novel role of NCoR1 in impairing spatial memory through the mediation of a novel interacting protein DEC2
Source: Cell Mol Life Sci. 2024 Jun 20;81(1):273. doi: 10.1007/s00018-024-05321-0 (PMC11335199; doi:10.1007/s00018-024-05321-0)
Supplement: Supplementary file 1 — Supplementary file1 (PDF 20 kb) [file 18_2024_5321_MOESM1_ESM.pdf]

Supplementary Figure Legends

Figure S1. The swim speed and locomotor activity are similar between *NCoR1 loxP* mice and *NCoR1* cKO mice

(A) The swim speed ($t_{1,16}$ = 0.95, $p > 0.05$) and **(B)** Visible platform learning performance ($F_{1,16}$ = 0.7, $p > 0.05$) of *NCoR1 loxP* mice and *NCoR1* cKO mice. N = 9 each group from the same animals in Figure 2. **(C)** Number of crossovers ($t_{1,10}$ = 0.24, $p > 0.05$) and mean movement speed ($t_{1,10}$ = 0.18, $p > 0.05$) for locomotor activity measure from a different batch of *NCoR1 loxP* mice and *NCoR1* cKO mice. N = 6 each group. **(D)** NCoR1 expression level in the hippocampus of *NCoR1 loxP* mice and *NCoR1* cKO mice from (C) ($t_{1,10}$ = 4.87, $p < 0.001$). Data are expressed as individual values and mean±SEM or expressed as mean±SEM (for B). *** $p < 0.001$.

Figure S2. NMDA administration decreases the association between NCoR1 and DEC2 and spatial training decreases the association between NCoR1 and HDAC3

(A) Rats received acute PBS or NMDA (8 mM) injection to their hippocampal CA1 area and their CA1 tissue lysate was subjected to immunoprecipitation using anti-NCoR1 antibody and immunoblotting using anti-DEC2 antibody and anti-NCoR1 antibody at 1 h later (right panel). These tissue lysates were also

subjected to western blot determination of NCoR1 expression (right-lower panel). For the control experiment, the same tissue lysates were subjected to immunoprecipitation with IgG and immunoblotting with anti-DEC2 antibody (left panel). **(B)** The quantified result of (A) for the association between NCoR1 and DEC2 ($t_{1,6} = 9.44$, $p < 0.001$) and between NCoR1 and NCoR1 ($t_{1,6} = 10.53$, $p < 0.001$) (N = 4 each group). **(C)** The CA1 tissue from trained and non-trained rats was subjected to immunoprecipitation using anti-HDAC3 antibody and immunoblotting using anti-NCoR1 antibody. The same tissue lysates were immunoprecipitated with IgG and immunoblotted with anti-NCoR1 antibody to serve as the control experiment. These tissue lysates were also subjected to western blot determination of HDAC3 expression (lower panel). **(D)** The quantified result of (C) for the association between HDAC3 and NCoR1 ($t_{1,4} = 5.8$, $p < 0.01$) and for HDAC3 expression in the hippocampal lysate ($t_{1,4} = 0.07$, $p > 0.05$) (N = 3 each group). **(E)** DEC2 expression level was examined in trained and non-trained animals and a representative gel pattern is shown. **(F)** The quantified result of (E) ($t_{1,8} = 12.93$, $p < 0.001$). Data are expressed as individual values and mean±SEM. ** $p < 0.01$ and *** $p < 0.001$.

Figure S3. *DEC2* siRNA transfection enhances but DEC2WT plasmid transfection impairs spatial learning and memory performance

(A) Mice received control siRNA or *DEC2* siRNA (15 pmol) transfection to their hippocampal CA1 area and they were subjected to water maze learning 48 h later for two consecutive days with four trials a day ($F_{1,12} = 26.09$, $p < 0.001$). N = 7 each group. **(B)** Probe trial performance (time spent in the target quadrant) ($t_{1,12} = 2.22$, $p < 0.05$) and representative swim patterns from the same animals. **(C)** Total distance traveled in the target quadrant from the same animals ($t_{1,12} = 4.66$, $p < 0.001$). **(D)** Swim speed ($t_{1,12} = 0.03$, $p > 0.05$) for the probe trial test from the same animals. **(E)** DEC2 expression level in the hippocampus from the same animals after the probe trial test ($t_{1,12} = 11.26$, $p < 0.001$). **(F)** Mice received Flag-vector or Flag-DEC2WT plasmid transfection to their hippocampal CA1 area and they were subjected to water maze learning 48 h later for two consecutive days with four trials a day ($F_{1,12} = 37.23$, $p < 0.001$). N = 7 each group. **(G)** Probe trial performance (time spent in the target quadrant) ($t_{1,12} = 2.77$, $p < 0.05$) and representative swim patterns from the same animals. **(H)** Total distance traveled in the target quadrant from the same animals ($t_{1,12} = 2.31$, $p < 0.05$). **(I)** Swim speed ($t_{1,12} = 0.36$, $p > 0.05$) for the probe trial test from the same animals. **(J)** DEC2 expression level in the hippocampus from the same animals after the probe trial test ($t_{1,12} = 11.83$, $p < 0.001$). The same tissue lysates were immunoprecipitated with anti-Flag antibody and immunoblotted with anti-Flag antibody to confirm the transfection and expression of the Flag-DEC2WT plasmid

(lower panel). Data are expressed as individual values and mean±SEM or expressed as mean±SEM (for A and E). * $p < 0.05$ and *** $p < 0.001$.

Figure S4. Transfection efficiency in primary culture and Neuro2A cells

(A) Immunofluorescence staining of EGFP (green) and DAPI (blue) after pcDNA3-EGFP plasmid transfection to mouse primary neurons is shown. **(B)** Number of EGFP-positive neurons over total number of neurons is calculated as the transfection efficiency. N=4. **(C)** Immunofluorescence staining of EGFP (green) and DAPI (blue) after pcDNA3-EGFP plasmid transfection to Neuro2A cells is shown. **(D)** Number of EGFP-positive neurons over total number of neurons is calculated as the transfection efficiency. N=4. Individual values are shown.
