# Supplementary figures and images for: Novel role of NCoR1 in impairing spatial memory through the mediation of a novel interacting protein DEC2
Source: Cell Mol Life Sci. 2024 Jun 20;81(1):273. doi: 10.1007/s00018-024-05321-0 (PMC11335199; doi:10.1007/s00018-024-05321-0)

# Supplementary Figure 1

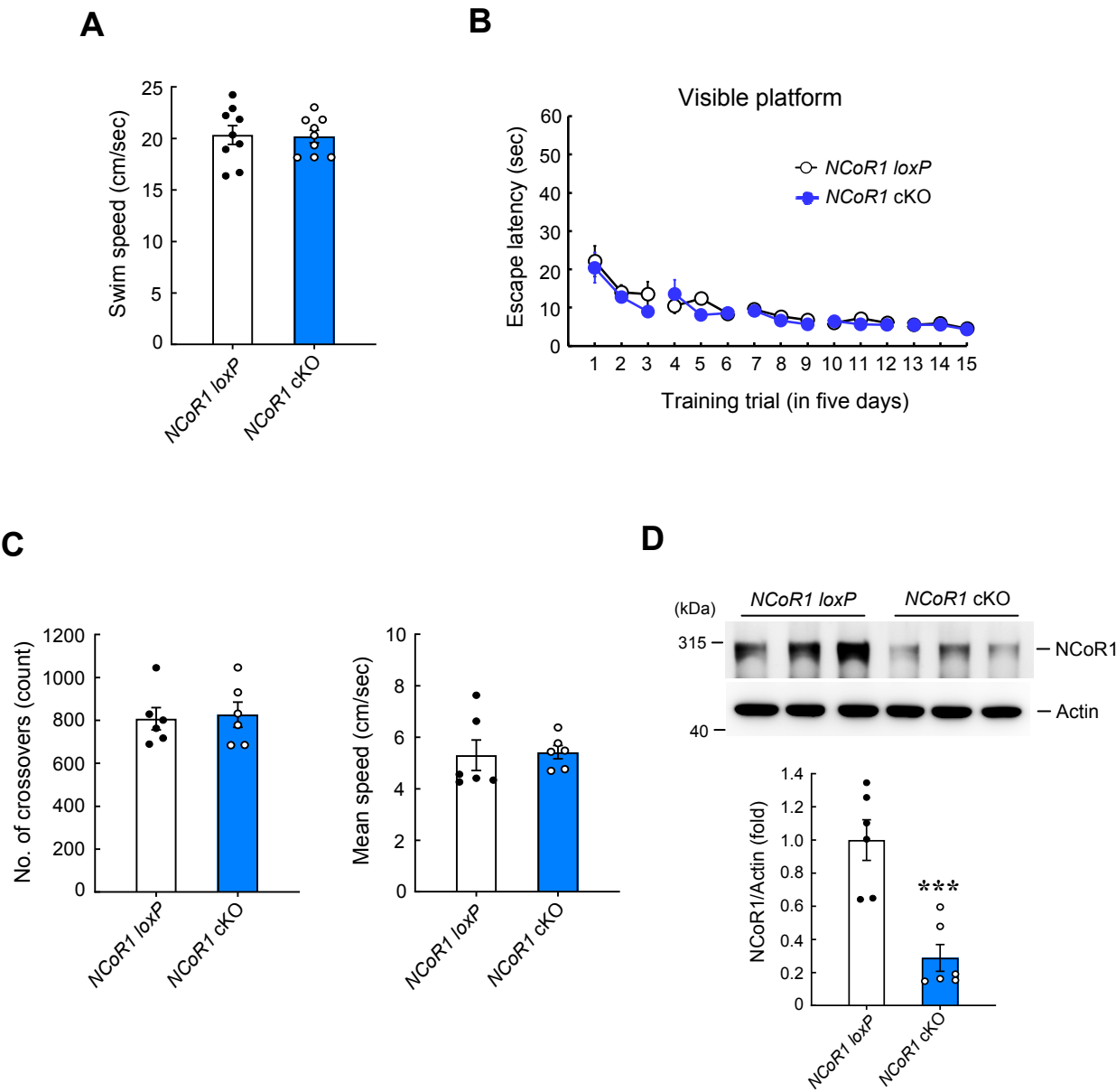

Supplement: Supplementary file 2 — Supplementary file2 (PDF 114 kb) [file 18_2024_5321_MOESM2_ESM.pdf]

# Supplementary Figure 2

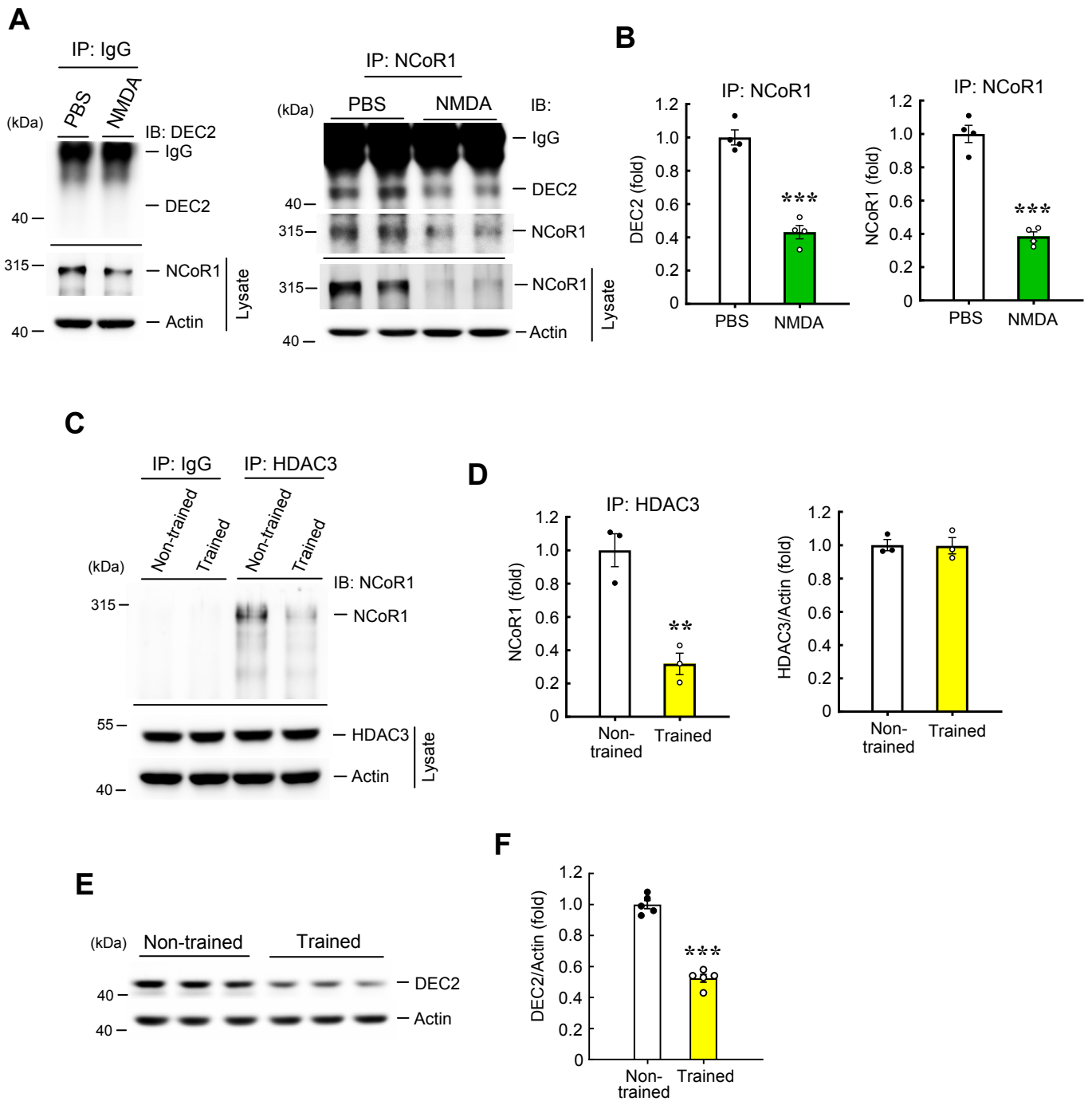

Supplement: Supplementary file 3 — Supplementary file3 (PDF 190 kb) [file 18_2024_5321_MOESM3_ESM.pdf]

# Supplementary Figure 3

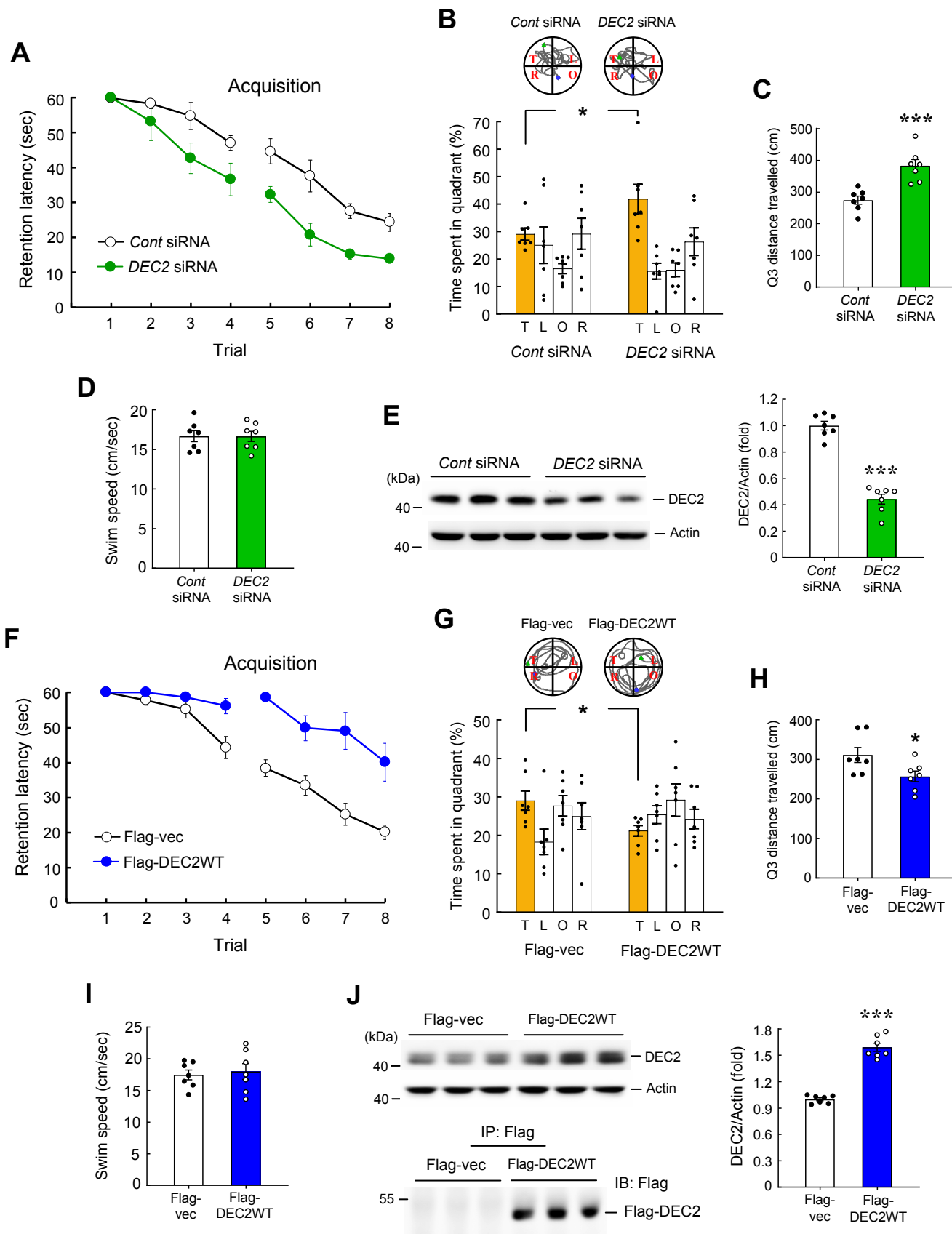

Supplement: Supplementary file 4 — Supplementary file4 (PDF 170 kb) [file 18_2024_5321_MOESM4_ESM.pdf]
