# Supplementary material for: Novel role of NCoR1 in impairing spatial memory through the mediation of a novel interacting protein DEC2
Source: Cell Mol Life Sci. 2024 Jun 20;81(1):273. doi: 10.1007/s00018-024-05321-0 (PMC11335199; doi:10.1007/s00018-024-05321-0)

# Supplementary Figure 4

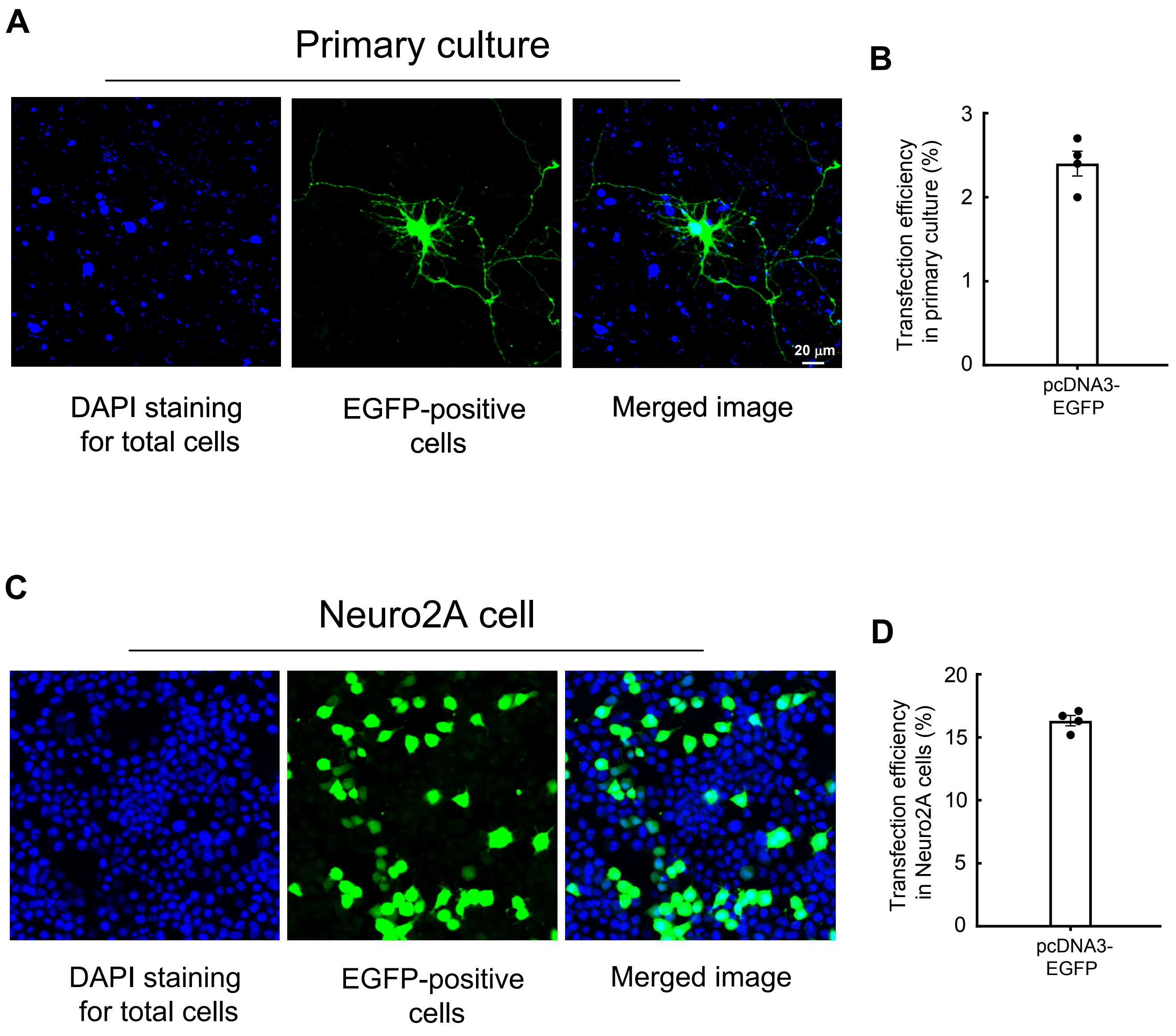

Supplement: Supplementary file 5 — Supplementary file5 (PDF 627 kb) [file 18_2024_5321_MOESM5_ESM.pdf]
