# Supplementary material for: Novel role of NCoR1 in impairing spatial memory through the mediation of a novel interacting protein DEC2
Source: Cell Mol Life Sci. 2024 Jun 20;81(1):273. doi: 10.1007/s00018-024-05321-0 (PMC11335199; doi:10.1007/s00018-024-05321-0)

Supplementary Figure 5 (original blots for all figures)

Figure 1

A

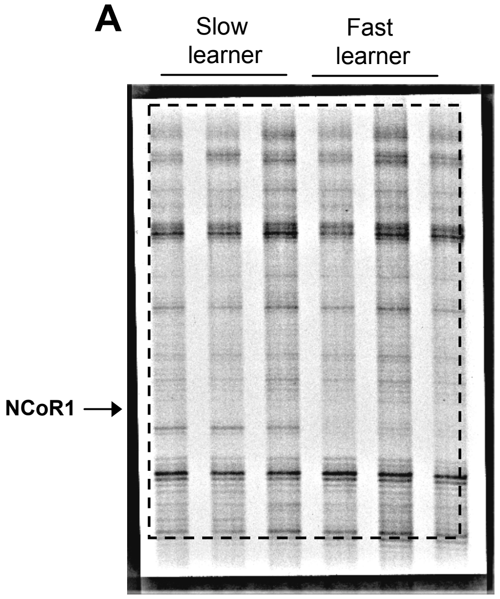

E

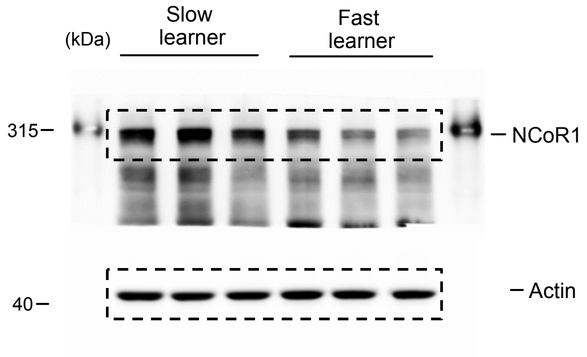

G

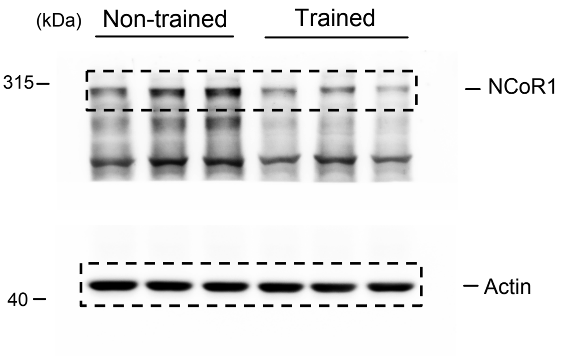

Figure 2

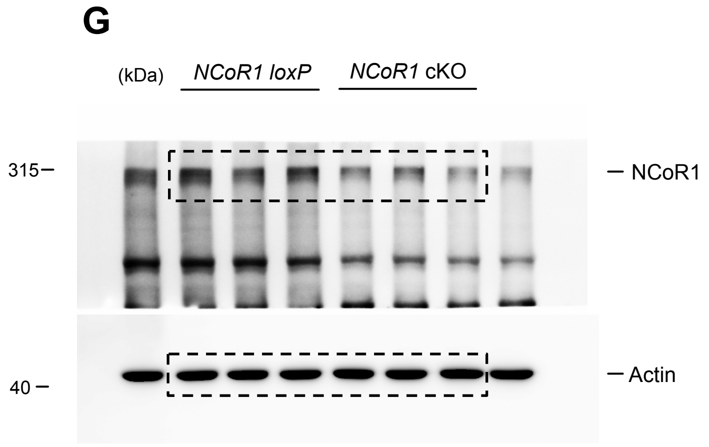

Figure 3

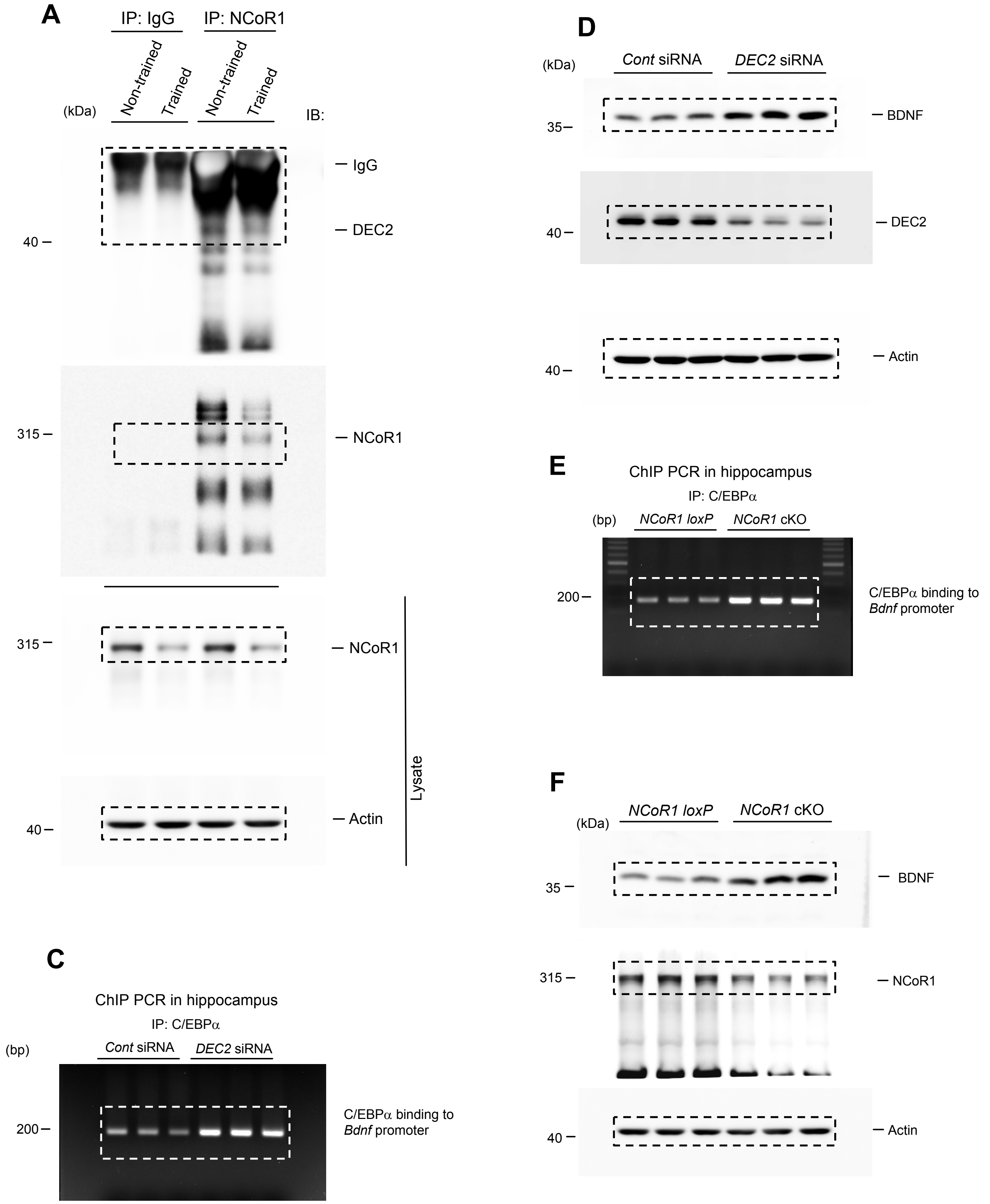
A
IP: IgG
IP: NCoR1
Non-trained
Trained
Non-trained
Trained
(kDa)
IB:
IgG
DEC2
40
315
NCoR1
315
NCoR1
Lysate
40
Actin
C
ChIP PCR in hippocampus
IP: C/EBPα
(bp)
Cont siRNA
DEC2 siRNA
200
C/EBPα binding to
Bdnf promoter
D
(kDa)
Cont siRNA
DEC2 siRNA
35
BDNF
40
DEC2
40
Actin
E
ChIP PCR in hippocampus
IP: C/EBPα
(bp)
NCoR1 loxP
NCoR1 cKO
200
C/EBPα binding to
Bdnf promoter
F
(kDa)
NCoR1 loxP
NCoR1 cKO
35
BDNF
315
NCoR1
40
Actin

Figure 4

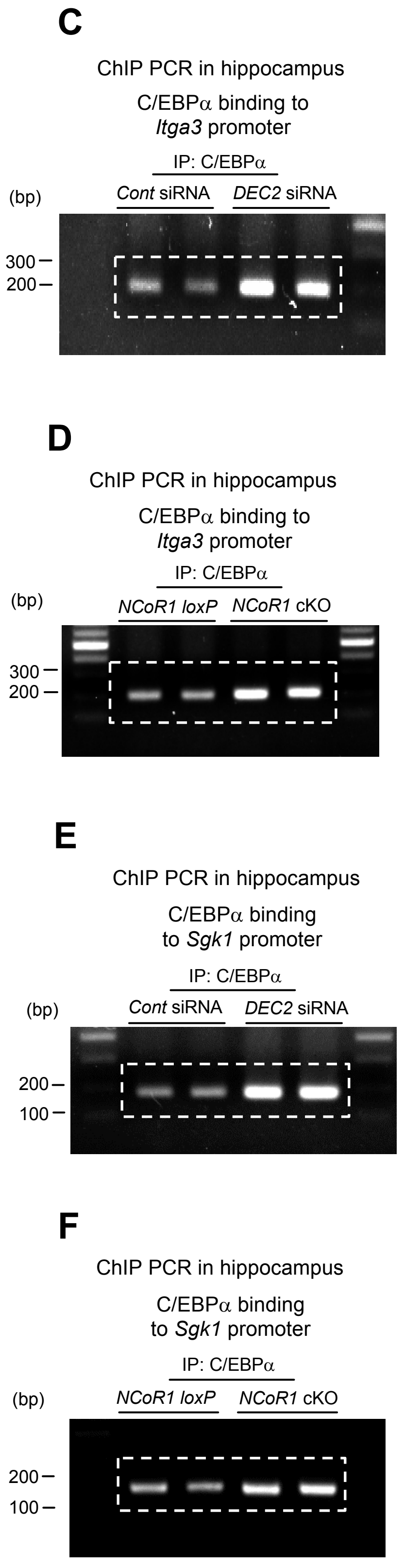

C
ChIP PCR in hippocampus
C/EBPα binding to
Itga3 promoter
IP: C/EBPα
Cont siRNA
DEC2 siRNA
(bp)
300
200
D
ChIP PCR in hippocampus
C/EBPα binding to
Itga3 promoter
IP: C/EBPα
NCoR1 loxP
NCoR1 cKO
(bp)
300
200
E
ChIP PCR in hippocampus
C/EBPα binding
to Sgk1 promoter
IP: C/EBPα
Cont siRNA
DEC2 siRNA
(bp)
200
100
F
ChIP PCR in hippocampus
C/EBPα binding
to Sgk1 promoter
IP: C/EBPα
NCoR1 loxP
NCoR1 cKO
(bp)
200
100

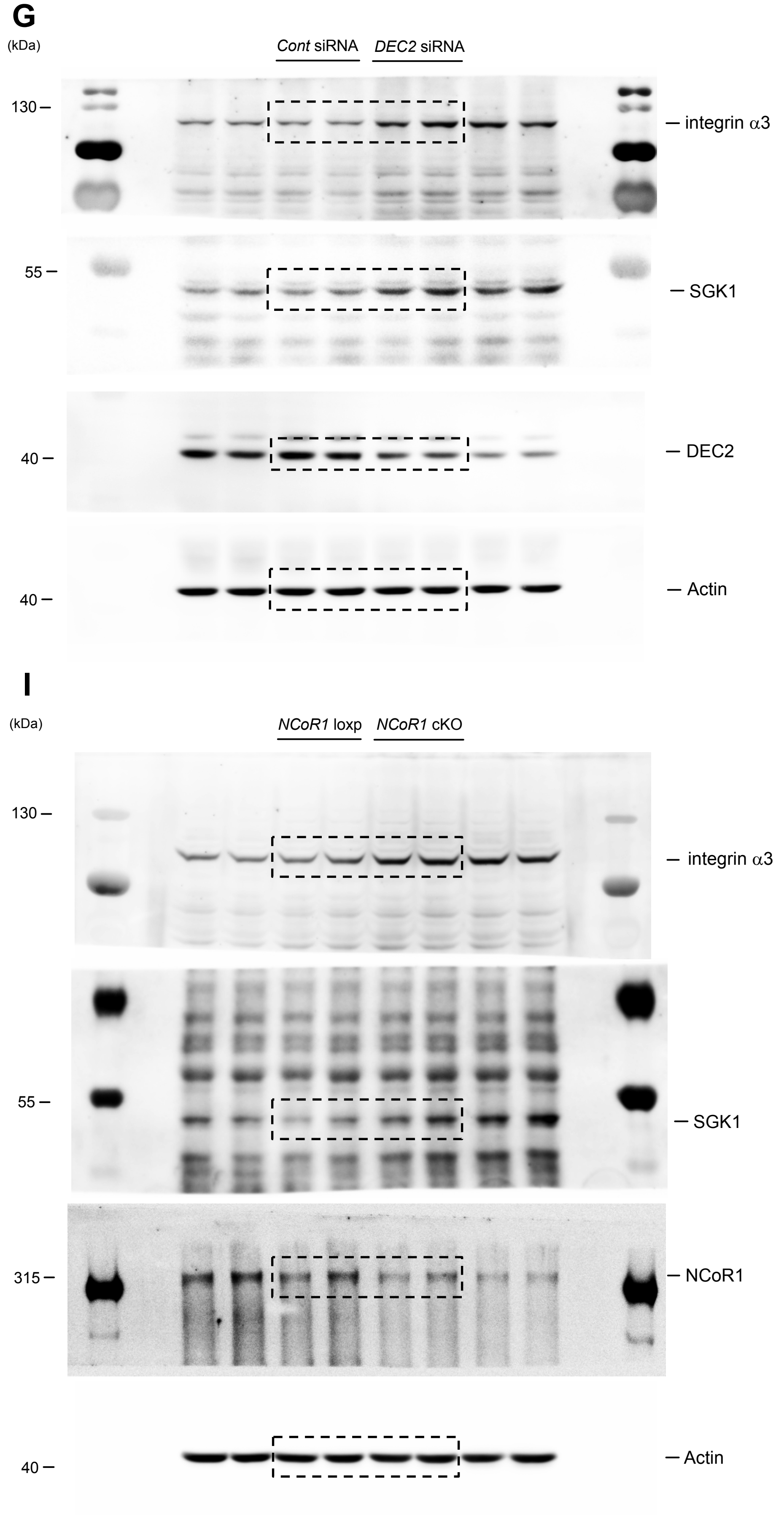

G
(kDa)
Cont siRNA
DEC2 siRNA
130
integrin α3
55
SGK1
40
DEC2
40
Actin
I
(kDa)
NCoR1 loxp
NCoR1 cKO
130
integrin α3
55
SGK1
315
NCoR1
40
Actin

Figure 5

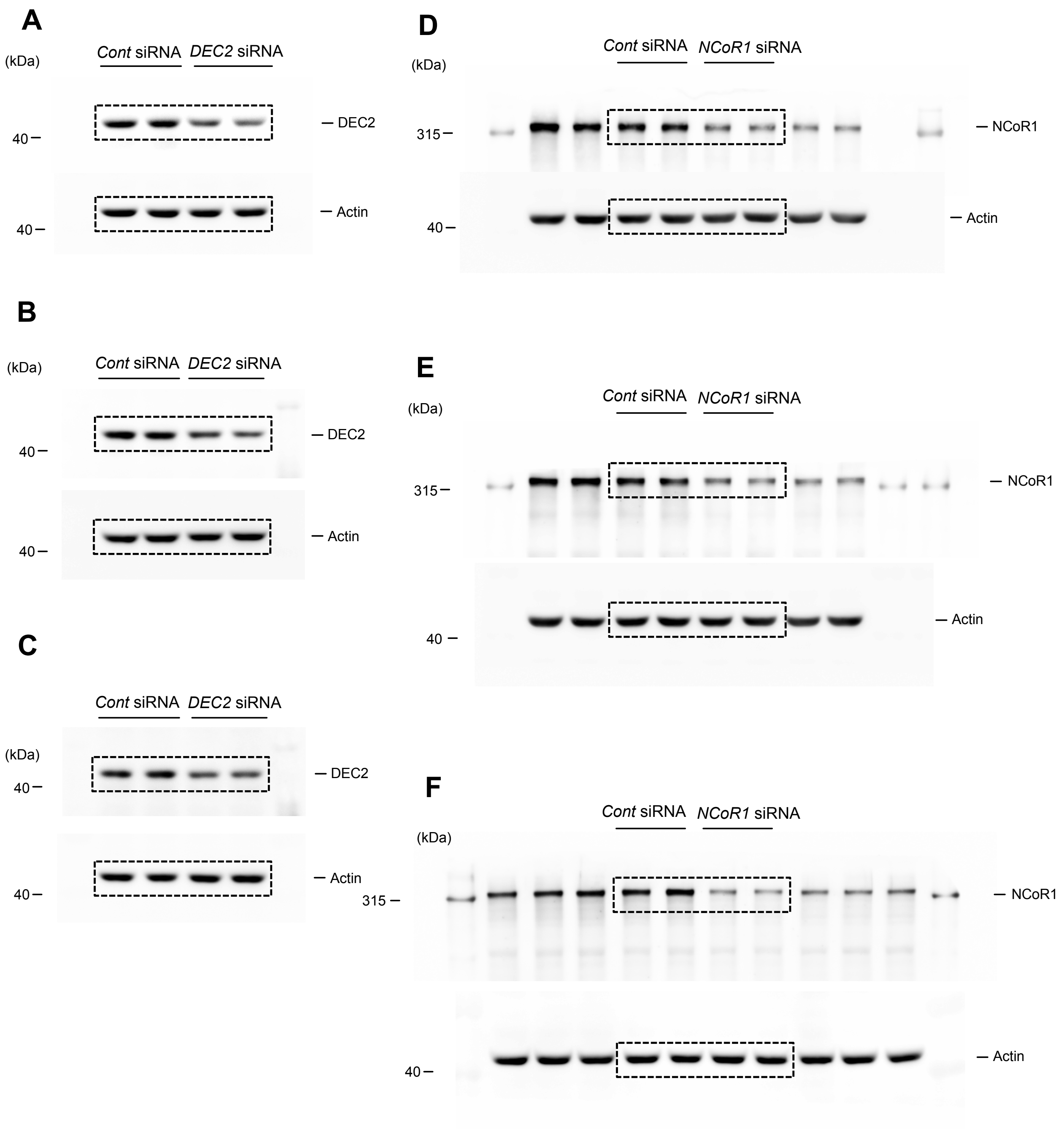
A
Cont siRNA DEC2 siRNA
(kDa)
40
DEC2
40
Actin
B
Cont siRNA DEC2 siRNA
(kDa)
40
DEC2
40
Actin
C
Cont siRNA DEC2 siRNA
(kDa)
40
DEC2
40
Actin
D
Cont siRNA NCoR1 siRNA
(kDa)
315
NCoR1
40
Actin
E
Cont siRNA NCoR1 siRNA
(kDa)
315
NCoR1
40
Actin
F
Cont siRNA NCoR1 siRNA
(kDa)
315
NCoR1
40
Actin

Figure 6

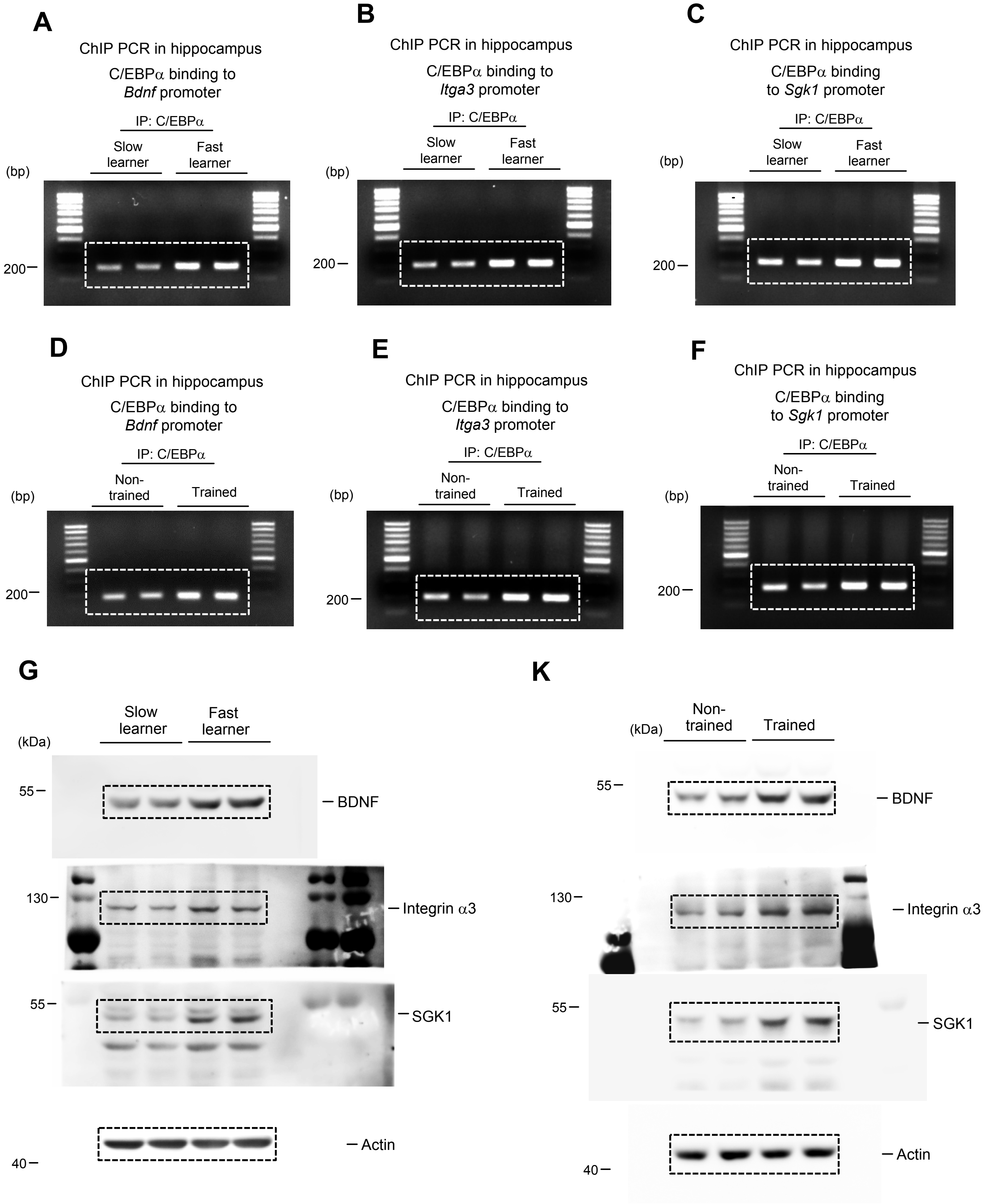

A
ChIP PCR in hippocampus
C/EBPα binding to
Bdnf promoter
IP: C/EBPα
Slow learner
Fast learner
(bp)
200
B
ChIP PCR in hippocampus
C/EBPα binding to
Itga3 promoter
IP: C/EBPα
Slow learner
Fast learner
(bp)
200
C
ChIP PCR in hippocampus
C/EBPα binding
to Sgk1 promoter
IP: C/EBPα
Slow learner
Fast learner
(bp)
200
D
ChIP PCR in hippocampus
C/EBPα binding to
Bdnf promoter
IP: C/EBPα
Non-trained
Trained
(bp)
200
E
ChIP PCR in hippocampus
C/EBPα binding to
Itga3 promoter
IP: C/EBPα
Non-trained
Trained
(bp)
200
F
ChIP PCR in hippocampus
C/EBPα binding
to Sgk1 promoter
IP: C/EBPα
Non-trained
Trained
(bp)
200
G
Slow learner
Fast learner
(kDa)
55
BDNF
130
Integrin α3
55
SGK1
Actin
40
K
Non-trained
Trained
(kDa)
55
BDNF
130
Integrin α3
55
SGK1
Actin
40

Figure 7

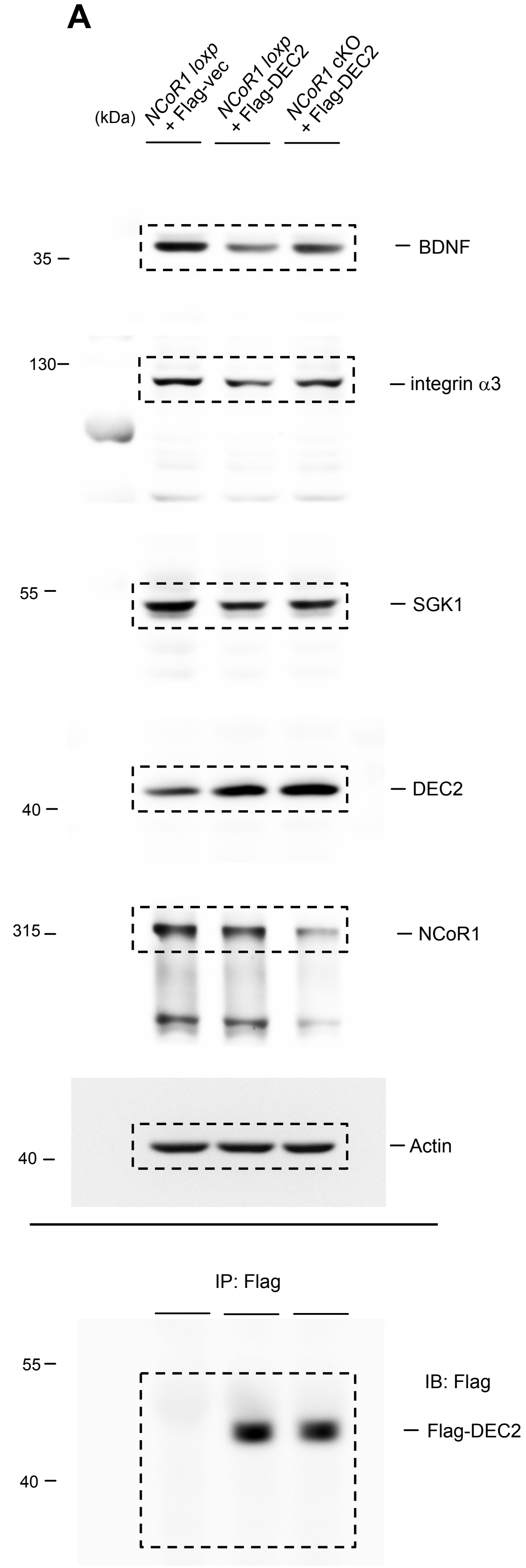
A
(kDa)
NCoR1 loxp + Flag-vec
NCoR1 loxp + Flag-DEC2
NCoR1 cKO + Flag-DEC2
35
BDNF
130
integrin α3
55
SGK1
DEC2
40
315
NCoR1
40
Actin
IP: Flag
55
IB: Flag
Flag-DEC2
40

Figure 8

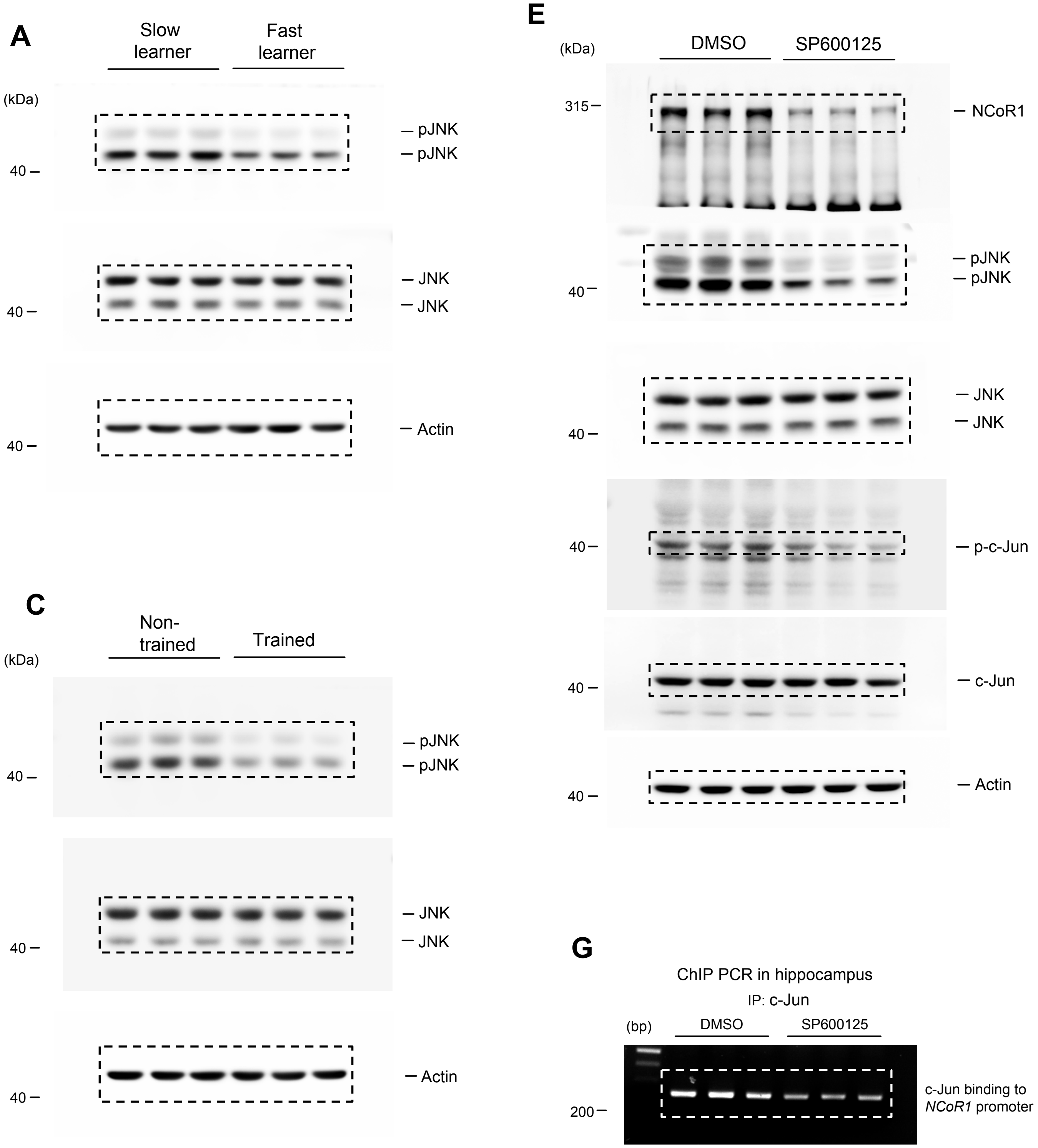

A
Slow learner
Fast learner
(kDa)
pJNK
pJNK
40
JNK
JNK
40
Actin
40
C
Non-trained
Trained
(kDa)
pJNK
pJNK
40
JNK
JNK
40
Actin
40
E
(kDa)
DMSO
SP600125
315
NCoR1
pJNK
pJNK
40
JNK
JNK
40
p-c-Jun
40
c-Jun
40
Actin
40
G
ChIP PCR in hippocampus
IP: c-Jun
(bp)
DMSO
SP600125
200
c-Jun binding to
NCoR1 promoter

Figure 10

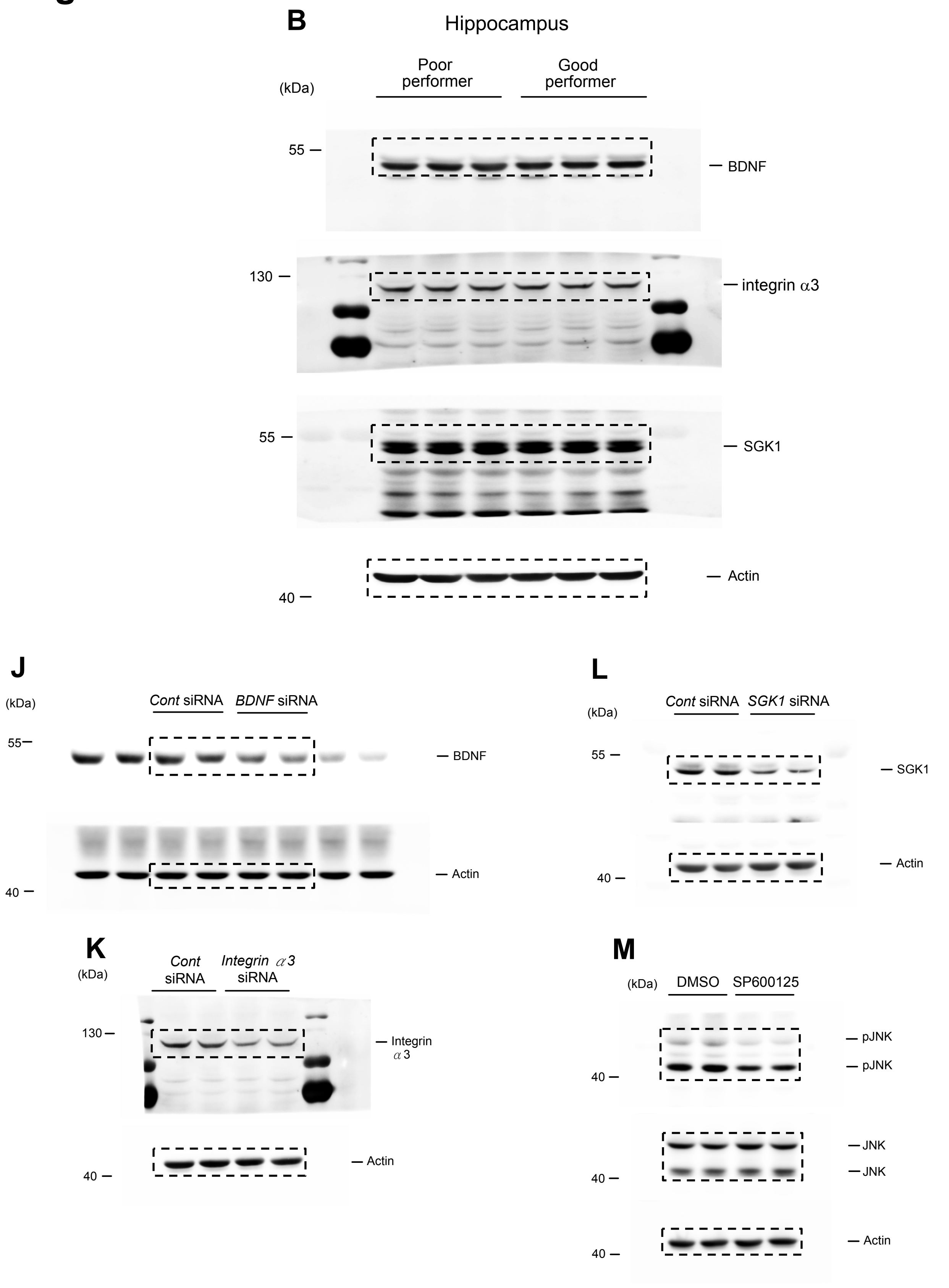
B
Hippocampus
Poor performer
Good performer
(kDa)
55
BDNF
130
integrin α3
55
SGK1
Actin
40
J
(kDa)
Cont siRNA
BDNF siRNA
55
BDNF
Actin
40
L
(kDa)
Cont siRNA
SGK1 siRNA
55
SGK1
Actin
40
K
(kDa)
Cont siRNA
Integrin α3 siRNA
130
Integrin α3
Actin
40
M
(kDa)
DMSO
SP600125
pJNK
pJNK
40
JNK
JNK
40
Actin
40

Supplementary Figure 1

D

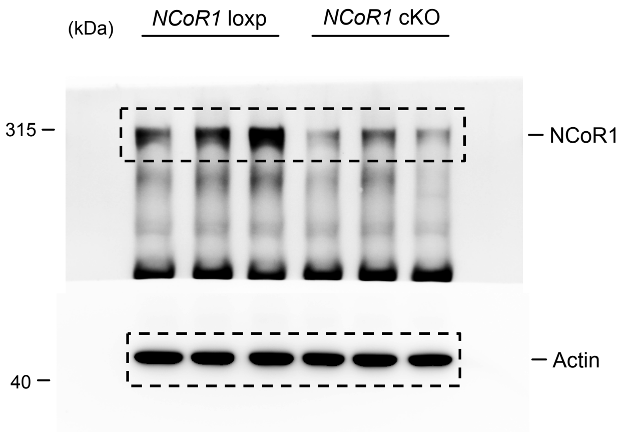

Supplementary Figure 2

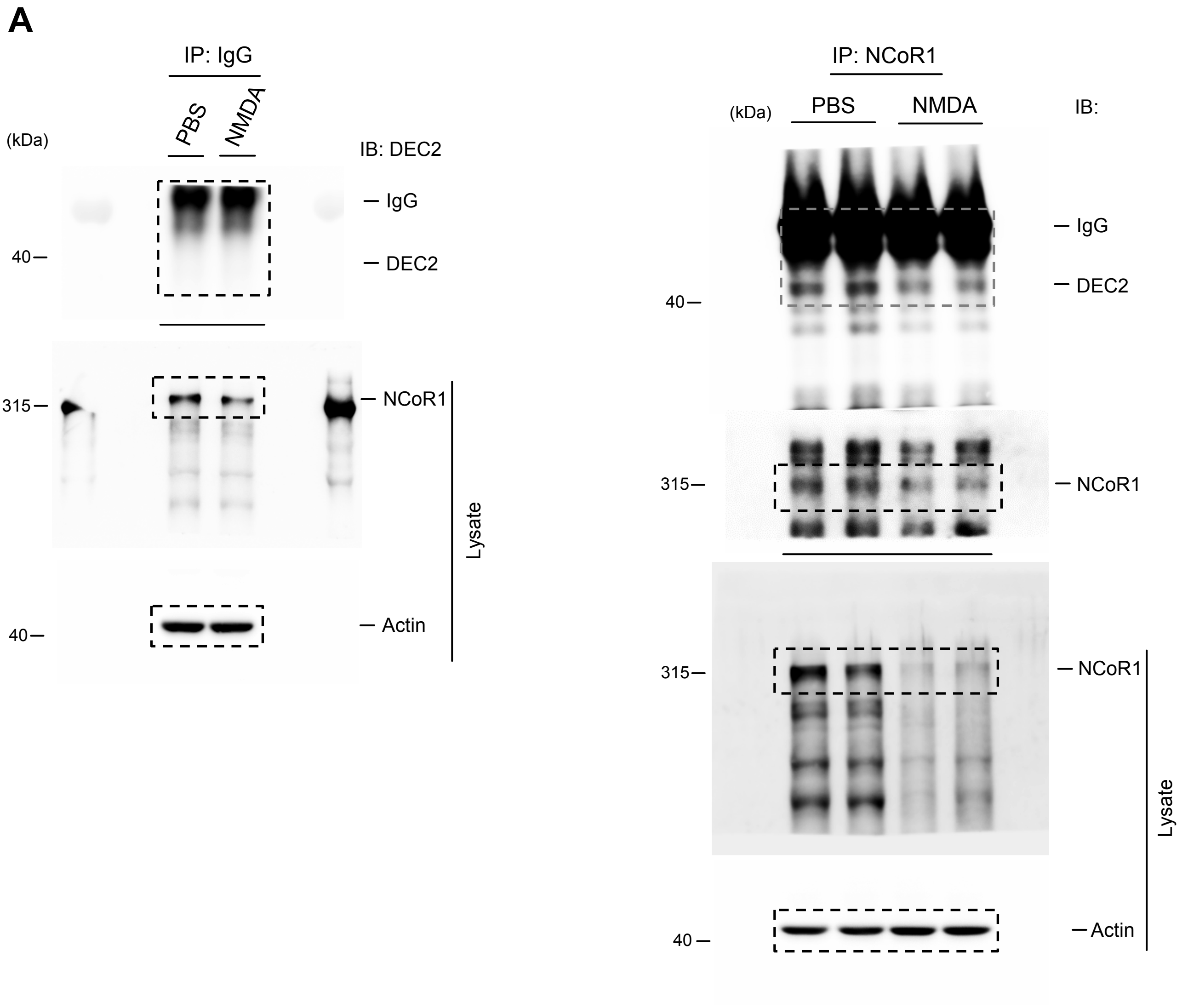

Supplementary Figure 2 (continue)

C

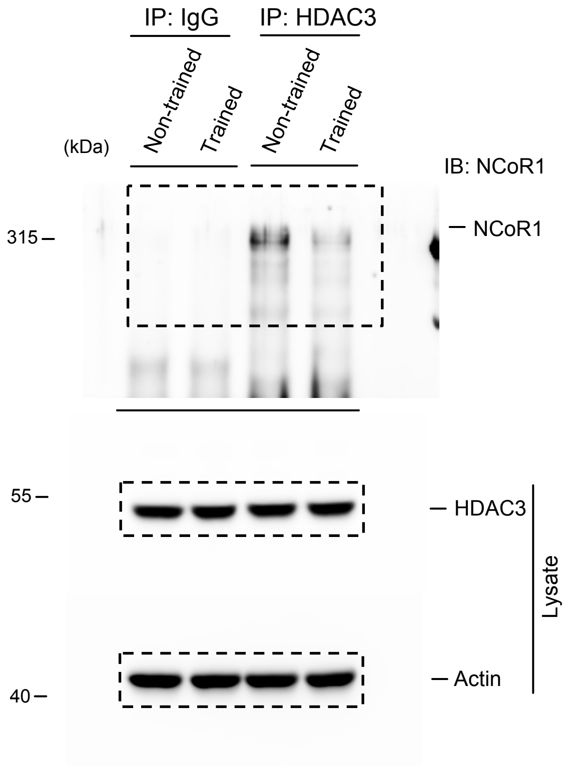

E

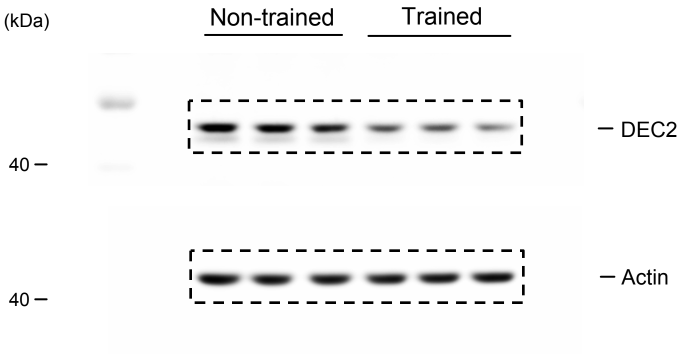

Supplementary Figure 3

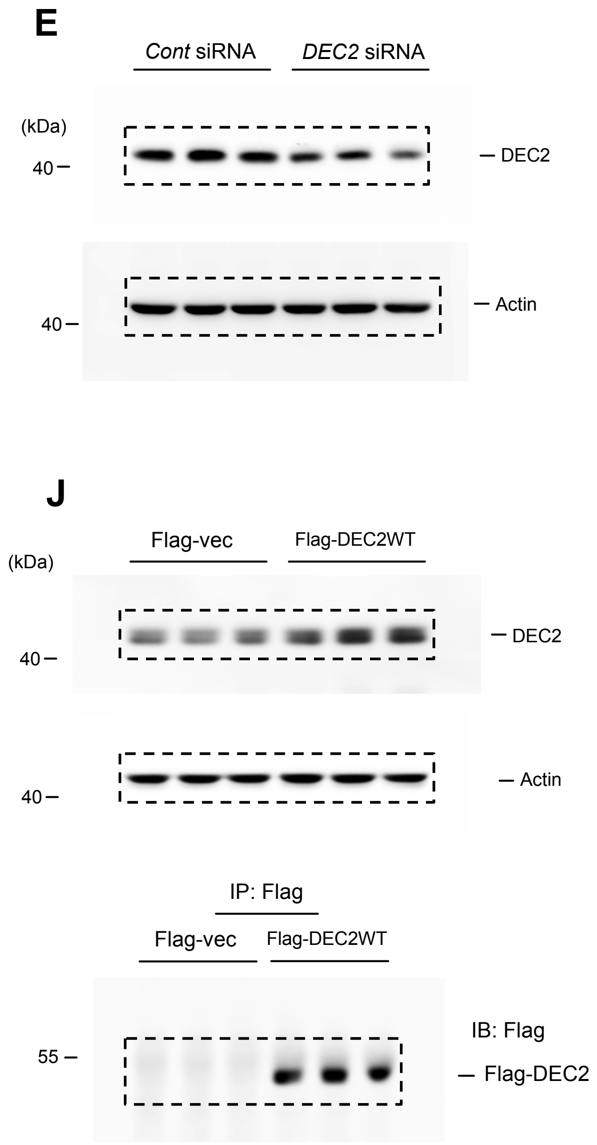

Supplement: Supplementary file 6 — Supplementary file6 (PDF 3872 kb) [file 18_2024_5321_MOESM6_ESM.pdf]
